# Predicting lymph node metastasis in non-small cell lung cancer using clinical and PET/CT-derived morphological features: An interpretable machine-learning study

**DOI:** 10.1371/journal.pone.0361043

**Published:** 2026-10-15

**Authors:** Yunchao Xu, Jiajun Hao, Yujiao Dou, Biao Zeng

**Affiliations:** 1 The Affiliated Cancer Hospital of Xiangya School of Medicine, Central South University/Hunan Cancer Hospital, Changsha, Hunan, People’s Republic of China; 2 Department of Radiological Diagnosis, The 970 Hospital of the Joint Logistics Support Force of the People’s Liberation Army of China, Yantai City, Shandong Province, China; 3 Department of Clinical Nursing, Xiangya Hospital, Central South University, Changsha, China; Fondazione Policlinico Universitario Agostino Gemelli IRCCS, ITALY

## Abstract

**Background:**

Accurate prediction of lymph node metastasis (LNM) in non-small cell lung cancer (NSCLC) is crucial for staging and treatment planning. This study aimed to establish a standardized machine learning workflow based on routinely available clinical, **serological,** and PET/CT-derived parameters to enhance preoperative LNM risk stratification.

**Methods:**

This retrospective study included 255 patients with histologically confirmed NSCLC, all of whom underwent systematic lymph-node dissection. Patients were randomly divided, with stratification by pathological lymph-node status, into a training cohort (n = 178) and an internal held-out test test cohort (n = 77). Candidate predictors were screened using univariable and multivariable logistic regression in the training cohort. Six machine-learning algorithms were trained and optimized using repeated nested five-fold cross-validation. After model development was completed, all six models were applied without modification to the test cohort. Test-set AUCs were compared using paired DeLong tests with Holm correction. The primary model was further evaluated for calibration, clinical utility, and feature contribution using SHAP. A conventional multivariable logistic regression model using the same four predictors was added as a simple reference model, and its discrimination and calibration were compared with those of XGBoost.

**Results:**

Central tumor location, elevated carcinoembryonic antigen, elevated squamous cell carcinoma antigen, and solid nodule status were retained as predictors; none of the quantitative PET metabolic parameters remained independently associated with LNM. XGBoost achieved the highest cross-validated AUC among the six candidate machine-learning algorithms in the training cohort (0.735; 95% CI, 0.653–0.813) and was therefore designated the primary model. In the internal held-out test cohort, XGBoost achieved an AUC of 0.747 (95% CI, 0.631–0.846). The conventional logistic regression reference model achieved a test AUC of 0.733 (95% CI, 0.612–0.841), with no significant difference from XGBoost (DeLong P = 0.301). Its Brier score was 0.152, compared with 0.198 for XGBoost.

**Conclusion:**

XGBoost model showed moderate discrimination and a high negative predictive value for pathological LNM in NSCLC. However, its limited positive predictive value and the absence of significant differences among the candidate algorithms support cautious interpretation. External multicenter evaluation is required before clinical implementation.

## Introduction

Non-small cell lung cancer (NSCLC) remains the leading cause of cancer-related mortality worldwide, with the prognosis largely dependent on accurate staging at diagnosis [1].. Adenocarcinoma and squamous cell carcinoma together represent the vast majority of NSCLC [2,3]. The presence or absence of lymph node metastasis, particularly the involvement of N1 or N2 nodes, critically determines the staging, therapeutic strategies, and survival outcomes for NSCLC patients [4].

Among preoperative non-invasive diagnostic methods, ^18^F-fluorodeoxyglucose positron emission tomography/computed tomography (^18^F-FDG PET/CT) is widely considered the most reliable imaging technique for evaluating mediastinal and hilar lymph node status in NSCLC. By assessing both metabolic activity and anatomical features, ^18^F-FDG PET/CT significantly enhances the accuracy of lymph node staging and informs clinical decision-making [5]. Metabolic parameters derived from ^18^F-FDG PET/CT, such as maximum standardized uptake value (SUVmax), metabolic tumor volume (MTV), and total lesion glycolysis (TLG), have been shown to play an important role in predicting lymph node metastasis in NSCLC. These quantitative indices provide objective measures of tumor metabolic activity, offering additional prognostic information beyond conventional imaging features and thereby improving the accuracy of preoperative risk stratification [6,7]. However, whether these quantitative PET indices retain independent predictive value after adjustment for morphological and clinical characteristics remains uncertain.

Machine learning (ML) has demonstrated remarkable capabilities in predicting clinical outcomes and supporting diagnostic decisions across various medical domains [8,9]. Unlike traditional statistical methods, ML algorithms can automatically identify complex, non-linear relationships and novel variable interactions within high-dimensional datasets, without relying on explicit programming or pre-defined assumptions. This allows for more accurate and individualized risk assessment, particularly in the context of cancer metastasis prediction. With its rapid evolution, ML is increasingly being applied in healthcare to enhance the precision of prognosis, diagnosis, and patient stratification.

SHapley Additive exPlanations (SHAP) is an advanced interpretability framework designed to provide detailed explanations for the predictions of any machine learning model. Rooted in cooperative game theory, SHAP leverages Shapley values to quantify the contribution of each feature to the model’s output [10]. In many studies, the lack of model interpretability has limited the clinical applicability of machine learning, as traditional approaches may fail to capture the complex relationships among variables or offer actionable insights for clinical decision-making. By employing SHAP, researchers can achieve both model transparency and clinically meaningful explanations, thereby facilitating the adoption of ML-based tools in healthcare.

Therefore, this study evaluated routinely available clinical, serological, and PET/CT-derived candidate features and developed an interpretable machine-learning framework for LNM risk prediction in patients with NSCLC.

## Materials and methods

### Patients

The study included NSCLC patients who underwent ^18^F‑FDG PET/CT scans between September 2017 and April 2024 at the Department of Nuclear Medicine. Retrospective data access and collection for this study were performed between 25/07/2026 and 01/08/2026. This study was approved by the Ethics Committee of Hunan Cancer Hospital (approval number: KYJJ‑2026‑238). **Inclusion criteria were as follows: [**1**]** Patients with histologically confirmed adenocarcinoma or squamous cell carcinoma [2], preoperative ^18^ F-FDG PET/ CT scans performed (with the interval between PET/CT scans and surgery or biopsy being less than 3 weeks), and [3] complete clinical and pathological data. Exclusion criteria included: [1] a preoperative history of other malignancies [2] poor image quality, and [3] patients who had received chemotherapy or radiation before undergoing PET/CT. A total of 255 patients met the study criteria. All included patients underwent systematic lymph-node dissection, and no patients assessed solely by lymph-node sampling or biopsy were included. CEA and SCC antigen were recorded as normal or elevated according to the institutional laboratory reference ranges applicable at the time of measurement. The original continuous concentrations were not available for the present analysis.

We collected clinical, pathological, and imaging data from the hospital’s electronic medical record system for all eligible patients. Collected variables included age, sex, smoking history, previous tumor history, family history, and histological type (adenocarcinoma or squamous cell carcinoma). Tumor markers were recorded, including carcinoembryonic antigen (CEA), carbohydrate antigen 125 (CA125), carbohydrate antigen 15−3 (CA15−3), carbohydrate antigen 19−9 (CA19−9), alpha-fetoprotein (AFP), and squamous cell carcinoma antigen (SCC).

CT-based imaging features encompassed the long diameter, short diameter, and margin morphology of the tumor. CT attenuation was measured by placing a region of interest on the maximum axial cross-section of the tumor while avoiding visible necrosis, vessels, and calcification, and the mean attenuation value in Hounsfield units was recorded. Metabolic parameters derived from PET/CT included the maximum and mean standardized uptake values (SUVmax and SUVmean), metabolic tumor volume (MTV), and total lesion glycolysis (TLG), which was computed using the standard formula: TLG = SUVmean × MTV. Tumor location was categorized as central if the lesion lay within the inner third of the lung parenchyma (extending from the hilum to the pleura); otherwise, it was classified as peripheral. For the classification of nodule type, we used the consolidation-to-tumor ratio (CTR), defined as the ratio of the maximum consolidation diameter measured on the mediastinal window to the maximum tumor diameter measured on the lung window. Nodules were classified into four types according to CTR values: a CTR value of 1 was defined as a solid nodule (Solid), a value of 0 as a pure ground-glass nodule (pGGO), a value between 0 and 0.5 as a ground-glass-predominant nodule (GGO-P), and a value between 0.5 and 1 as a solid-predominant nodule (Solid-P). The gold standard for confirming LNM was histopathology. The reference standard for LNM was postoperative histopathological examination of the systematically dissected lymph nodes. Patients with pathological N1 or N2 metastasis were classified as LN-positive, whereas those without pathological N1 or N2 metastasis were classified as LN-negative.

### PET/CT image acquisition

All imaging was performed using a GE Discovery MI PET/CT scanner (GE Healthcare). Before ^18^F-FDG injection, patients fasted for at least 6 hours, and blood glucose levels were confirmed to be below 11 mmol/L. Subsequently, ^18^F-FDG was administered intravenously at a dose of 0.10 mCi/kg (radiochemical purity >95%). After an uptake period of 45–60 minutes, whole-body PET/CT imaging was performed. CT images were acquired at a tube voltage of 110 kV using a helical acquisition protocol with a pitch of approximately 1.0 and were reconstructed with a matrix size of 512 × 512 and a slice thickness of approximately 3–4 mm. The PET acquisition lasted approximately 12 minutes, and the images were reconstructed using the vendor-provided QCFX-S 450 algorithm with a matrix of 192 × 192. CT data were used for PET attenuation correction.

### Data processing

Before feature screening and data preprocessing, the complete dataset was randomly divided at the patient level into a training cohort and an internal held-out test cohort at a 7:3 ratio, with stratification by pathological lymph-node status. The training cohort included 178 patients, whereas the test cohort included 77 patients. Univariable and multivariable logistic regression analyses were subsequently performed using only the training cohort. Variables with P < 0.05 in the univariable analyses were entered simultaneously into the multivariable model, and variables retaining P < 0.05 were used for machine-learning development. This procedure identified four binary predictors: central tumor location, elevated CEA, elevated SCC antigen, and solid nodule status.

The cohort was imbalanced, with LNM present in 58 of 255 patients (22.7%). Because all four selected predictors were binary, we used the Synthetic Minority Over-sampling Technique for Nominal variables (SMOTEN) rather than conventional SMOTE. SMOTEN generates synthetic minority-class observations from neighboring categorical feature patterns without interpolating continuous values. Oversampling was performed only within each cross-validation training fold. The independent test cohort was not oversampled and did not contribute to preprocessing estimates or threshold selection; it was used only for model evaluation.

### Model construction and evaluation

Six machine-learning classifiers were evaluated using the same four predictors identified in the training cohort: support vector machine (SVM), artificial neural network (ANN), XGBoost, extra trees, decision tree, and gradient boosting. Model development was performed using repeated nested five-fold cross-validation with three repeats. Hyperparameters were optimized within the inner cross-validation folds. The classifier with the highest cross-validated AUC in the training cohort was designated as the primary model. Each classifier was then optimized using the complete training cohort, finalized, and applied without modification to the internal held-out test cohort. For each model, the classification cutoff was determined from its cross-validated training predictions using the Youden index and fixed before test-cohort evaluation. For comparison, a conventional multivariable logistic regression model containing exactly the same four predictors was fitted as a simple reference model without additional feature selection or oversampling. Its training performance was estimated using repeated stratified five-fold cross-validation with three repeats. The final logistic regression model was fitted to the original complete training cohort and applied without modification to the internal held-out test cohort. Its operating cutoff was determined from the cross-validated training predictions using the Youden index and fixed before test evaluation. The logistic reference model was not used to alter the training-based selection of XGBoost as the primary machine-learning model. Discrimination was assessed using receiver operating characteristic (ROC) curve analysis. AUCs and 95% confidence intervals were estimated using 10,000 patient-level bootstrap resamples. Additional performance measures included sensitivity, specificity, accuracy, positive predictive value, negative predictive value, F1 score, confusion matrices, and Brier score. Calibration was assessed graphically and by calculating the calibration intercept and slope, for which ideal values are 0 and 1, respectively; 95% confidence intervals were estimated using 3,000 patient-level bootstrap resamples. The test-cohort AUCs of XGBoost and conventional logistic regression were compared using a two-sided paired DeLong test. Decision-curve analysis was used to assess net clinical benefit across a range of threshold probabilities.

### Model interpretation

To enhance the interpretability of the machine learning model, SHapley Additive exPlanations (SHAP) was employed. SHAP provides both global and local interpretability by calculating the contribution of each feature to individual predictions, thereby improving model transparency and explainability.

Feature importance was visualized using summary plots, bar plots, waterfall plots, and force plots generated from SHAP values. These visualizations allowed for both global interpretation of feature impact across the entire dataset and detailed single-sample explanations, facilitating a comprehensive understanding of how specific variables influenced the model’s predictions.

### Statistical analysis

Statistical analysis was performed using R 4.4.0. Normality and variance homogeneity of continuous variables were checked via the Shapiro-Wilk and Levene tests, respectively. Data following a normal distribution are presented as mean ± SD; otherwise, medians with quartiles are reported. Comparisons between two groups were conducted using either the independent t-test or the Mann–Whitney U-test. The constituent ratios of count data were compared using a 2 × 2 chi-squared test (two-tailed). Significance was defined as p < 0.05. The conventional logistic regression reference model and calibration analyses were conducted in Python using statsmodels and scikit-learn.

## Results

### Patient characteristics

The 255 patients were stratified into a training cohort of 178 patients (40 LNM-positive, 22.5%) and a test cohort of 77 patients (18 LNM-positive, 23.4%). As shown in Table 1, the prevalence of LNM and the distributions of demographic, tumor, PET/CT, histological, and tumor-marker characteristics were comparable between the two cohorts, with no statistically significant baseline differences (all P > 0.05).

**Table 1:** Clinical characteristics of the patients.

Characteristic	Overall(n = 255)	Training(n = 178)	test(n=77)	*P value*
Lymph node status				1.000
LN-negative	197 (77.3)	138 (77.5)	59 (76.6)	
LN-positive	58 (22.7)	40 (22.5)	18 (23.4)	
Age, years	62.15 + /- 8.49	62.54 + /- 8.47	61.23 + /- 8.52	0.331
Long diameter, mm	26.00 [18.00, 36.00]	26.00 [18.00, 36.00]	26.00 [17.00, 36.00]	0.704
Short diameter, mm	18.00 [12.00, 27.00]	18.50 [13.00, 27.00]	15.00 [11.00, 26.00]	0.246
CT value, HU	26.00 [11.00, 37.00]	25.00 [10.00, 36.00]	27.00 [14.00, 37.00]	0.582
SUVmax	8.34 [3.74, 13.39]	8.21 [3.60, 12.82]	8.93 [3.84, 14.64]	0.513
SUVmean	4.13 [2.00, 6.05]	4.13 [2.00, 6.01]	4.00 [2.05, 6.20]	0.891
MTV	6.69 [2.91, 17.70]	6.76 [3.02, 18.14]	6.26 [2.77, 17.39]	0.837
TLG	25.05 [6.78, 101.17]	25.46 [6.43, 92.79]	23.15 [7.31, 108.15]	0.927
Sex				0.191
Female	115 (45.1)	75 (42.1)	40 (51.9)	
Male	140 (54.9)	103 (57.9)	37 (48.1)	
Smoking history				0.113
No	102 (40.0)	65 (36.5)	37 (48.1)	
Yes	153 (60.0)	113 (63.5)	40 (51.9)	
Tumor location				0.625
Peripheral	189 (74.1)	134 (75.3)	55 (71.4)	
Central	66 (25.9)	44 (24.7)	22 (28.6)	
Tumor margin				0.775
Smooth	184 (72.2)	127 (71.3)	57 (74.0)	
Rough	71 (27.8)	51 (28.7)	20 (26.0)	
Nodule type				0.826
pGGO	20 (7.8)	13 (7.3)	7 (9.1)	
GGO-predominant	21 (8.2)	14 (7.9)	7 (9.1)	
Solid-predominant	22 (8.6)	17 (9.6)	5 (6.5)	
Solid	192 (75.3)	134 (75.3)	58 (75.3)	
Histological type				0.586
Squamous cell carcinoma	59 (23.1)	39 (21.9)	20 (26.0)	
Adenocarcinoma	196 (76.9)	139 (78.1)	57 (74.0)	
CEA				0.375
Normal	198 (77.6)	135 (75.8)	63 (81.8)	
Elevated	57 (22.4)	43 (24.2)	14 (18.2)	
CA125				0.692
Normal	236 (92.5)	166 (93.3)	70 (90.9)	
Elevated	19 (7.5)	12 (6.7)	7 (9.1)	
CA15−3				0.221
Normal	242 (94.9)	171 (96.1)	71 (92.2)	
Elevated	13 (5.1)	7 (3.9)	6 (7.8)	
CA19−9				1.000
Normal	227 (89.0)	158 (88.8)	69 (89.6)	
Elevated	28 (11.0)	20 (11.2)	8 (10.4)	
AFP				0.556
Normal	252 (98.8)	175 (98.3)	77 (100.0)	
Elevated	3 (1.2)	3 (1.7)	0 (0.0)	
SCC antigen				0.694
Normal	217 (85.1)	153 (86.0)	64 (83.1)	
Elevated	38 (14.9)	25 (14.0)	13 (16.9)	

### Model performance

Training-cohort univariable and multivariable logistic regression results are shown in Table 2. Six variables met the univariable entry criterion (P < 0.05): central location, CT value, SUVmean, elevated CEA, elevated SCC antigen, and solid nodule status. After simultaneous multivariable adjustment, four variables remained independently associated with LNM: central location (OR 2.66, 95% CI 1.14–6.19; P = 0.023), elevated CEA (OR 3.07, 95% CI 1.33–7.10; P = 0.009), elevated SCC antigen (OR 2.91, 95% CI 1.08–7.88; P = 0.035), and solid nodule status (OR 9.26, 95% CI 1.11–77.10; P = 0.039). CT value and SUVmean were not independently significant after adjustment. None of the quantitative PET metabolic parameters, including SUVmax, SUVmean, MTV, and TLG, remained independently associated with LNM after multivariable adjustment.

**Table 2:** Results of logistic regression analysis for clinical characteristics.

Characteristic (reference level)	Univariate analysis			Multivariable analysis		
	OR	95%CI	*p-value*	OR	95%CI	*p-value*
Age, per year	1.00	0.96-1.04	0.936			
Sex (male vs female)	1.47	0.71-3.06	0.301			
Smoking (yes vs no)	1.99	0.90-4.39	0.089			
Location (central vs peripheral)	2.62	1.23-5.58	0.013*	2.66	1.14-6.19	0.023*
Margin (rough vs smooth)	1.27	0.59-2.71	0.541			
Long diameter, per mm	1.00	0.99-1.01	0.743			
Short diameter, per mm	1.03	1.00-1.06	0.052			
CT value, per HU	1.00	1.00-1.01	0.027*	1.00	1.00-1.01	0.393
SUVmax, per unit	1.01	0.99-1.03	0.452			
SUVmean, per unit	1.18	1.04-1.34	0.013*	1.02	0.86-1.21	0.832
MTV, per unit	1.00	0.99-1.01	0.942			
TLG, per unit	1.00	1.00-1.00	0.711			
Histology (adenocarcinoma vs squamous)	0.48	0.22-1.06	0.069			
CEA (elevated vs normal)	3.70	1.73-7.90	<0.001*	3.07	1.33-7.10	0.009*
CA125 (elevated vs normal)	2.67	0.80-8.94	0.110			
CA15−3 (elevated vs normal)	2.72	0.58-12.68	0.204			
CA19−9 (elevated vs normal)	2.63	0.99-6.96	0.052			
AFP (elevated vs normal)	1.74	0.15-19.74	0.653			
SCC antigen (elevated vs normal)	3.36	1.38-8.16	0.007*	2.91	1.08-7.88	0.035*
Nodule type (solid vs non-solid)	17.65	2.35-132.72	0.005*	9.26	1.11-77.10	0.039*

Data in parentheses are reference level; * Represents p < 0.05. OR, odds ratio; CI, confidence interval.

The four training-selected predictors were used in all six candidate machine-learning models. XGBoost achieved the highest training AUC (0.735, 95% CI 0.653–0.813), narrowly exceeding GradientBoosting (0.730), SVM (0.722), DecisionTree (0.721), ExtraTrees (0.711), and ANN (0.690). XGBoost was therefore selected as the final model using training information only, and its Youden cutoff of 0.478 was fixed before the test cohort was evaluated (Table 3; Fig 1). The conventional logistic regression reference model achieved an AUC of 0.722 (95% CI, 0.631–0.803) in the training cohort and 0.733 (95% CI, 0.612–0.841) in the internal held-out test cohort. At its training-derived cutoff of 0.271, the test-cohort accuracy, sensitivity, specificity, PPV, NPV, F1 score, and Brier score were 0.675, 0.778, 0.644, 0.400, 0.905, 0.528, and 0.152, respectively (Table 3). The test AUC did not differ significantly from that of XGBoost (AUC difference, 0.014; paired DeLong P = 0.301). Because this reference analysis was added after the primary model had been selected, it was used to evaluate the value of additional model complexity and did not alter the original training-based model-selection procedure.

**Table 3:** Summary of performance metrics for all machine learning models on the training and test sets.

Model	Cohort	AUC (95% CI)	Accuracy	Sensitivity	Specificity	PPV	NPV	F1 score	Brier score
XGBoost	Training	0.735 (0.653–0.813)	0.685	0.775	0.659	0.397	0.910	0.525	0.206
	Test	0.747 (0.631–0.846)	0.675	0.778	0.644	0.400	0.905	0.528	0.198
Gradient boosting	Training	0.730 (0.654–0.803)	0.702	0.825	0.667	0.418	0.929	0.555	0.207
	Test	0.747 (0.629–0.850)	0.675	0.778	0.644	0.400	0.905	0.528	0.197
Extra trees	Training	0.711 (0.632–0.792)	0.691	0.775	0.667	0.403	0.911	0.530	0.210
	Test	0.739 (0.630–0.843)	0.675	0.778	0.644	0.400	0.905	0.528	0.201
Decision tree	Training	0.721 (0.635–0.798)	0.697	0.775	0.674	0.408	0.912	0.534	0.206
	Test	0.739 (0.618–0.849)	0.675	0.778	0.644	0.400	0.905	0.528	0.201
SVM	Training	0.722 (0.639–0.794)	0.674	0.800	0.638	0.390	0.917	0.525	0.206
	Test	0.730 (0.601–0.845)	0.649	0.778	0.610	0.378	0.900	0.509	0.203
ANN	Training	0.690 (0.596–0.783)	0.652	0.675	0.645	0.355	0.873	0.466	0.195
	Test	0.718 (0.600–0.823)	0.662	0.778	0.627	0.389	0.902	0.519	0.199
Logistic regression	Training	0.722 (0.631–0.803)	0.697	0.800	0.667	0.410	0.920	0.542	0.150
	Test	0.733 (0.612–0.841)	0.675	0.778	0.644	0.400	0.905	0.528	0.152

**Fig 1:**
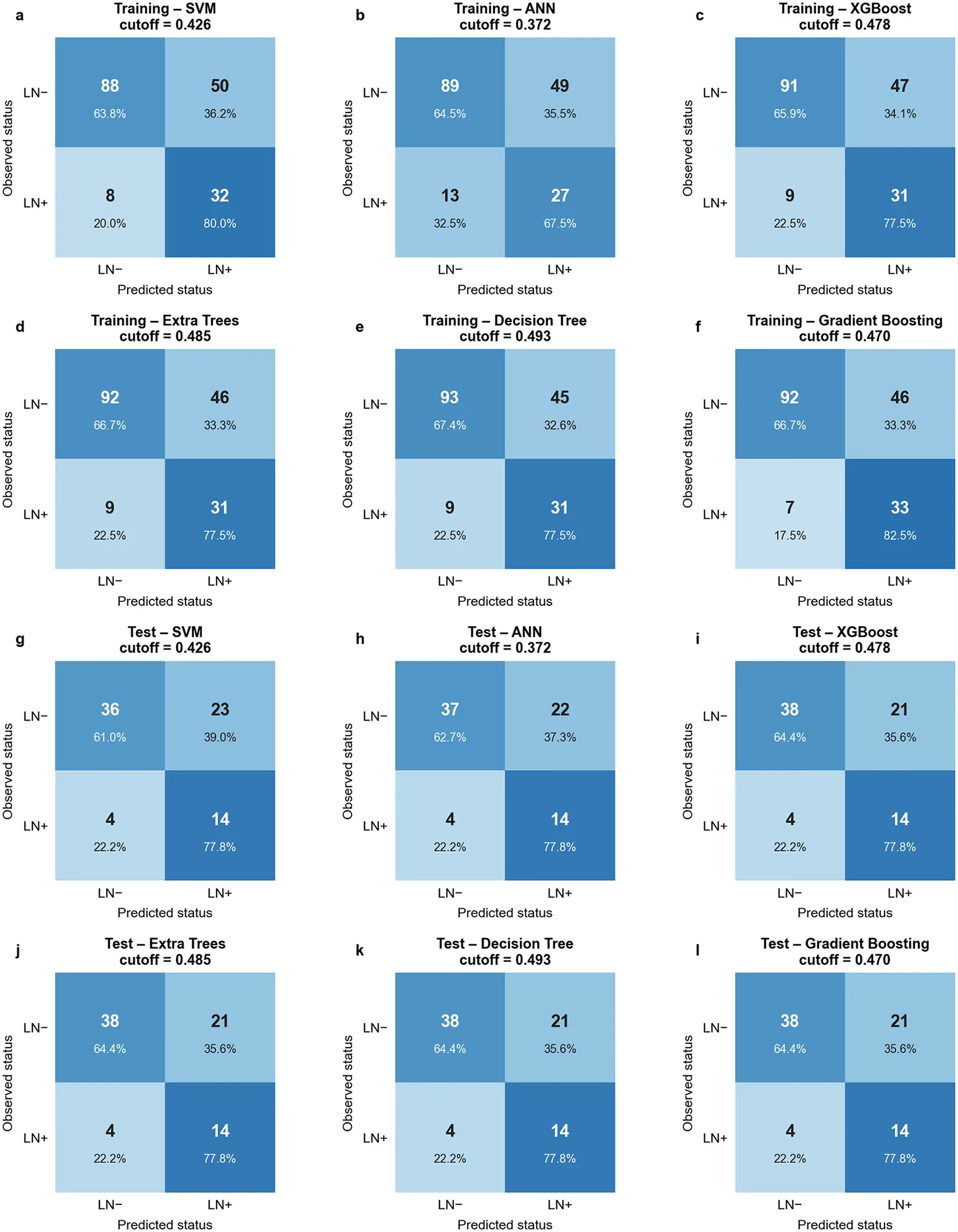
Confusion matrices for each model on the training and test sets.

In the internal held-out test cohort, the training-selected XGBoost model achieved an AUC of 0.747 (95% CI, 0.631–0.846) and an accuracy of 0.675. The sensitivity, specificity, PPV, NPV, F1 score, and Brier score were 0.778, 0.644, 0.400, 0.905, 0.528, and 0.198, respectively. Gradient boosting achieved the same test AUC of 0.747, whereas the AUCs of the remaining models ranged from 0.718 to 0.739. XGBoost had been selected solely according to its cross-validated AUC in the training cohort. Therefore, the test results were used only for independent evaluation and did not influence model selection.

Pairwise DeLong tests showed no statistically significant differences in test AUC among the six models after Holm correction (all adjusted P = 1.000). Accordingly, we do not claim that XGBoost significantly outperformed the other classifiers. Instead, XGBoost is reported as the primary model selected during training and subsequently evaluated in the internal held-out test cohort.

Precision–recall analysis further characterized model performance given the LNM prevalence of 23.4% in the internal held-out test cohort. Calibration analysis indicated deviations from ideal agreement across some probability ranges, although the overall Brier score was 0.198. Decision-curve analysis indicated potential net benefit across a limited range of low-to-intermediate threshold probabilities (Fig 2). These findings support cautious interpretation of the model as an adjunctive risk-stratification tool. In the internal held-out test cohort, the calibration intercept and slope were −1.140 (95% CI, −1.946 to −0.628) and 1.031 (95% CI, 0.568–1.930), respectively, for XGBoost, compared with 0.207 (95% CI, −0.649 to 1.278) and 1.095 (95% CI, 0.612–2.109), respectively, for conventional logistic regression. The logistic model also had a lower Brier score than XGBoost (0.152 vs 0.198), although uncertainty around the calibration estimates was substantial because of the modest test-cohort size (S1 Fig).

**Fig 2:**
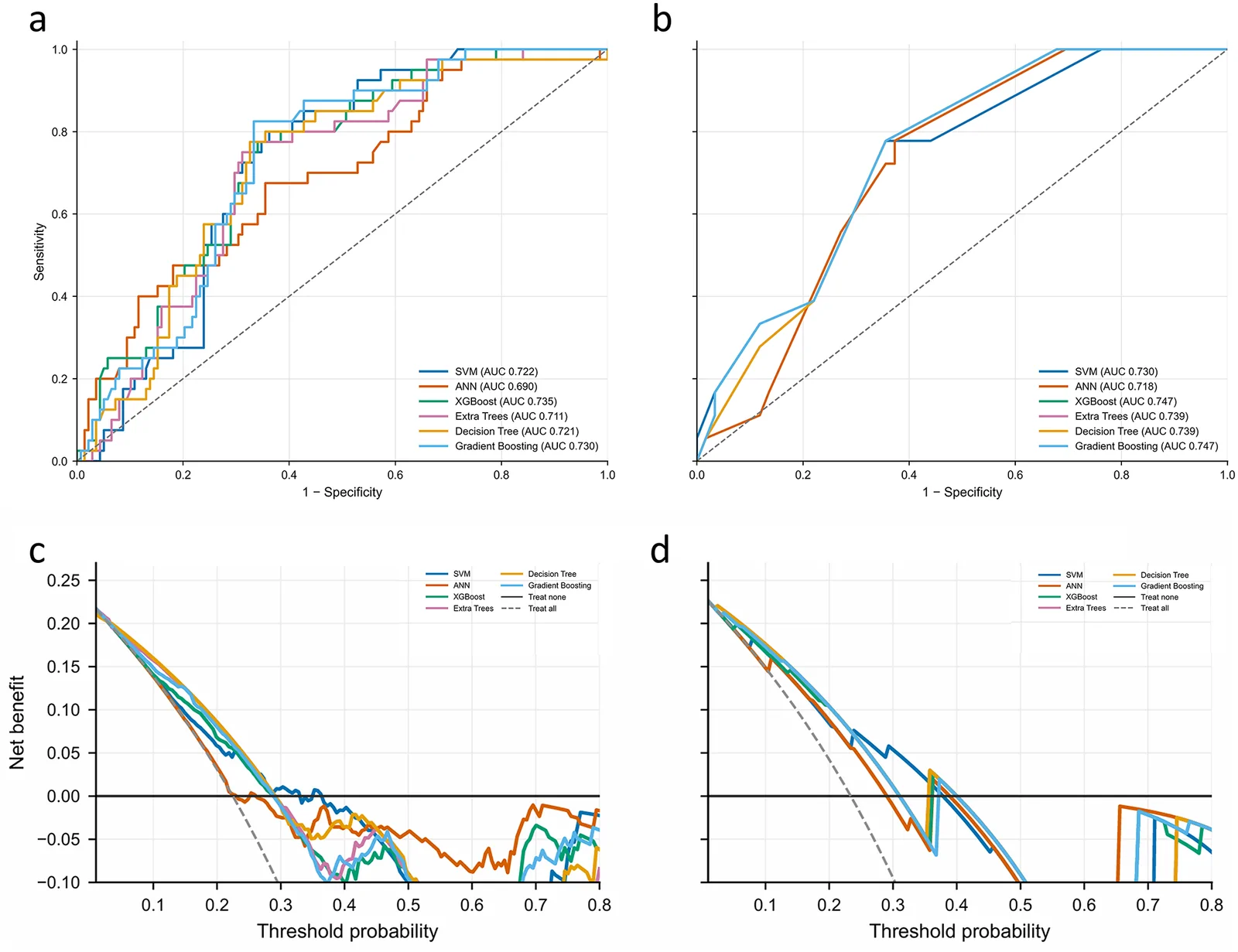
ROC curves and decision curve analysis (DCA) plots of each model on the training and test sets.

### Interpreting the model

SHAP analysis of the final XGBoost model showed that solid nodule status had the largest mean absolute SHAP value, indicating the greatest overall contribution to model predictions. Elevated CEA and central tumor location showed similar intermediate contributions, followed by elevated SCC antigen (Fig 3a).

**Fig 3:**
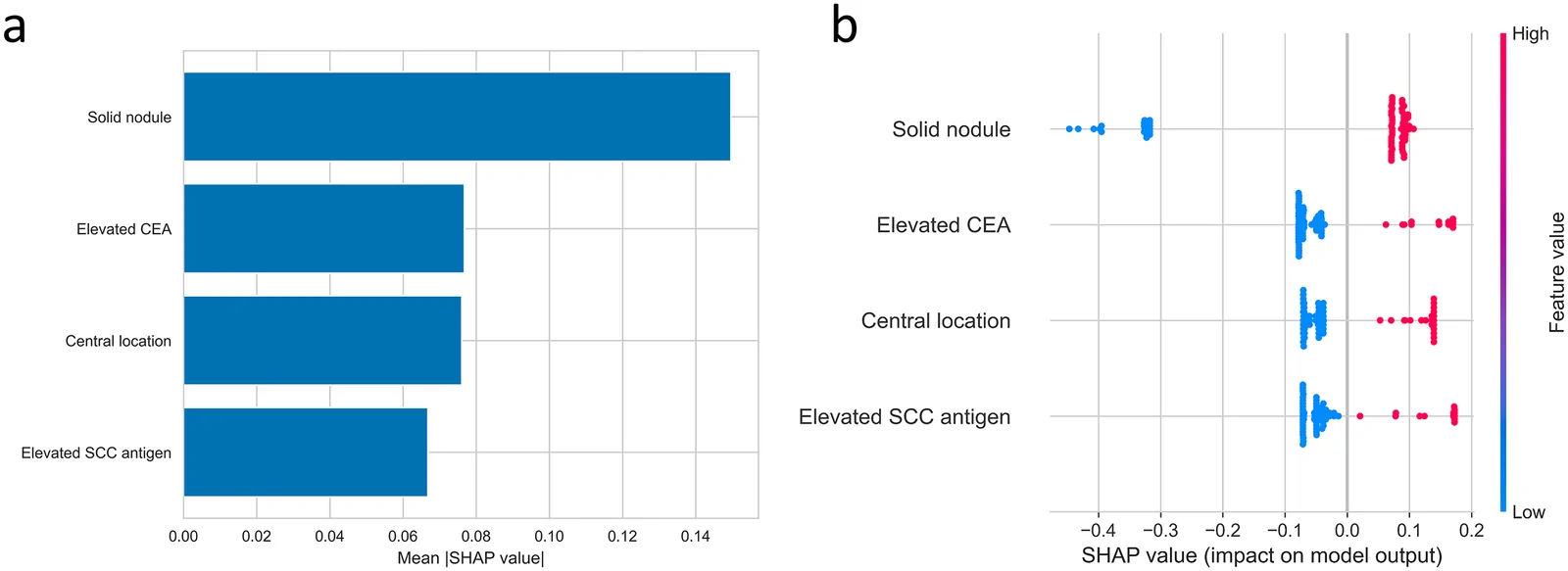
SHAP-based interpretation of feature importance in the XGBoost model. (**a**) Mean absolute SHAP values indicating the average contribution of each feature to the model output. (**b**) SHAP summary plot showing the direction and magnitude of each feature’s impact. Colors represent feature value (red: high, blue: low).

The SHAP beeswarm plot showed consistent directional patterns. Solid nodule status, elevated CEA, central tumor location, and elevated SCC antigen generally shifted predictions toward a higher probability of LNM. Conversely, the absence of these characteristics generally shifted predictions toward a lower probability (Fig 3b). The strong contribution of solid nodule status suggests that the model relied partly on a clinically recognizable high-risk imaging phenotype. However, the model should not be interpreted as a replacement for conventional nodal staging. The wide confidence interval for solid nodule status (OR, 9.26; 95% CI, 1.11–77.10) indicates uncertainty in the magnitude and stability of this association. Accordingly, its leading SHAP importance represents its contribution within the fitted XGBoost model and should not be interpreted as definitive evidence of a stable or causal clinical effect.

To better illustrate the clinical relevance of these features, two representative patients were selected for individualized SHAP interpretation (Fig 4). In Patient 1, a 63-year-old female, the model predicted a low-risk outcome.In contrast, Patient 2, a 61-year-old male was predicted as high risk.These visual explanations highlight how the model integrates clinical and imaging features to make case-specific predictions.

**Fig 4:**
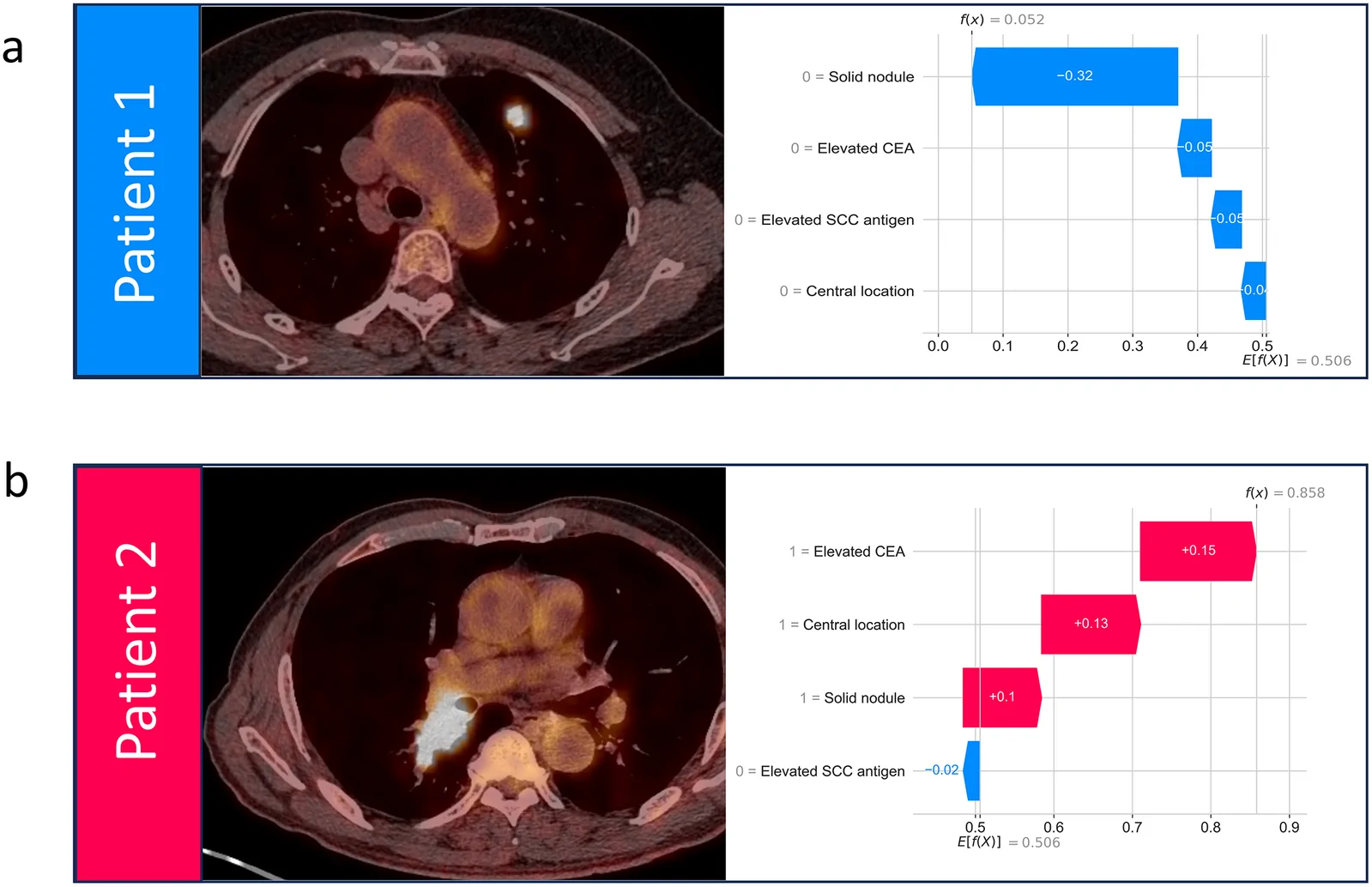
Patient-specific model interpretation using SHAP. (**a**) A 63-year-old female (Patient 1) with low predicted probability. (**b**) A 61-year-old male (Patient 2) with high predicted probability.

## Discussion

In this study, we developed an XGBoost-based predictive model incorporating four clinically and radiologically relevant predictors: central tumor location, elevated CEA, elevated SCC antigen, and solid nodule status. Although quantitative PET parameters were evaluated as candidate predictors, SUVmax, SUVmean, MTV, and TLG were not retained after multivariable adjustment. Thus, the final imaging contribution was derived primarily from CT-based morphological characteristics obtained during the PET/CT examination rather than from independent PET metabolic information. The model was developed to estimate the risk of LNM in patients with NSCLC.The model demonstrated moderate discriminatory performance in the internal held-out test cohort, with an AUC of 0.747 (95% CI, 0.631–0.846), sensitivity of 0.778, and specificity of 0.644. Compared with other machine learning algorithms, including SVM, ANN, decision tree, extra trees, and gradient boosting, XGBoost achieved the highest cross-validated AUC in the training cohort and was therefore selected as the primary model. However, gradient boosting achieved the same test AUC of 0.747, and pairwise DeLong tests showed no significant differences among the six models after Holm correction. Decision-curve analysis suggested potential net benefit only across a limited range of low-to-intermediate threshold probabilities. The conventional logistic regression model using the same four predictors achieved a similar test AUC of 0.733 (DeLong P = 0.301) and a lower Brier score than XGBoost (0.152 vs 0.198), providing no clear evidence that the additional complexity of XGBoost improved performance. The XGBoost NPV of 0.905 should be interpreted in the context of the test-cohort LNM prevalence of 23.4% and should not be considered evidence that the model can safely exclude nodal metastasis in clinically node-negative patients. In this study, we identified central tumor location, elevated CEA, elevated SCC antigen, and solid nodule status as independent predictive factors associated with LNM. These findings are in agreement with previous observations [8,11,12].

Despite the increasing application of machine learning in medical prediction tasks, many existing studies exhibit notable methodological limitations. Some rely on a single algorithm without conducting model comparison, which may compromise robustness and overlook potentially superior approaches [13,14]. In several cases, key preprocessing steps—such as data normalization or handling of missing values—are omitted or not clearly described, raising concerns about model stability and reproducibility [15,16]. Certain studies further lack rigorous validation strategies, failing to properly split training and testing datasets, or reporting results based solely on the entire cohort without external or internal validation [7,17]. The lack of explainability in black-box models also limits their adoption in clinical practice. Taken together, these issues hinder the reliability and translational value of prior models. Recent multicenter CT-based studies have evaluated radiomics and deep-learning models for occult LNM in early-stage lung adenocarcinoma using external cohorts [18,19]. Related multi-institutional studies in other malignancies have also examined explainable integration of PET/CT imaging and clinical data [20]. Because these studies differed in populations, outcomes, and input features, direct performance comparisons are not appropriate; however, they emphasize the importance of external validation. In contrast, our study employed a standardized modeling workflow, including stratified training-test splitting before feature selection, training-only feature screening, repeated nested cross-validation, preprocessing and oversampling confined to the training process, comparison of multiple classifiers, internal held-out test-cohort evaluation, and SHAP-based model interpretation. This study has several limitations. First, it was conducted retrospectively at a limited number of centers, which may introduce selection bias and limit the generalizability of the findings. Second, the sample size was relatively modest, and model performance was evaluated using an internal training-test split. External validation was not performed; therefore, further multicenter studies are required to confirm the robustness and generalizability of the model. The wide confidence interval for solid nodule status (OR, 9.26; 95% CI, 1.11–77.10) indicates uncertainty in the stability of this predictor; therefore, its SHAP ranking should be interpreted cautiously. Standardized clinical N category and node-level PET/CT assessments were not available, so the incremental value of the model beyond conventional clinical and PET/CT nodal staging could not be evaluated. Third, only a limited set of imaging and clinical features were included, and potentially valuable predictors such as genetic, pathological, or longitudinal data were not incorporated. Lastly, although SHAP was employed for model interpretation, further work is needed to integrate explainable AI into clinical decision workflows in a user-friendly and actionable manner.

## Conclusion

In conclusion, this study aimed to establish a standardized machine-learning workflow for LNM risk prediction using routinely available clinical, serological, and imaging characteristics. We developed an XGBoost-based model incorporating four binary predictors: central tumor location, elevated CEA, elevated SCC antigen, and solid nodule status. The model demonstrated moderate discriminatory performance in the internal held-out test cohort, while SHAP analysis clarified the relative contributions of the four predictors. Its AUC did not differ significantly from that of conventional logistic regression using the same predictors, and none of the quantitative PET metabolic parameters remained independently associated with LNM; the final imaging contribution was primarily morphological. These findings highlight the potential value of combining imaging characteristics and serological markers for individualized LNM risk stratification. However, the model should be regarded as an adjunctive risk-assessment tool rather than a replacement for conventional nodal staging. In the future, we plan to expand this framework by incorporating radiomics features and exploring multi-omics fusion approaches to further enhance predictive accuracy and support precision medicine applications.

## Supporting information

S1 Fig Calibration comparison between XGBoost and conventional multivariable logistic regression in the internal held-out test cohort. The diagonal line represents perfect calibration; points represent observed event proportions across groups of predicted probabilities.
(TIF)
