# Supplementary material for: Predicting lymph node metastasis in non-small cell lung cancer using clinical and PET/CT-derived morphological features: An interpretable machine-learning study
Source: PLoS One. 2026 Oct 15;21(10):e0361043. doi: 10.1371/journal.pone.0361043 (PMC13671544; doi:10.1371/journal.pone.0361043)
Supplement: Supplementary file 1 [file pone.0361043.s003.docx]

Response to Reviewers

**Manuscript ID:** PONE-D-26-39894
**Title:** Predicting lymph node metastasis in non-small cell lung cancer combining clinical and PET/CT features with machine learning: an interpretable SHAP-based study

**Dear Academic Editor and Reviewers,**

We thank the Academic Editor and Reviewers for their careful and constructive evaluation of our manuscript. We rebuilt the analysis from the original patient-level data using a leakage-free workflow. The patient-level training-test split was fixed before feature screening, and all feature selection, preprocessing, oversampling, hyperparameter tuning, algorithm ranking, and cutoff selection were confined to the training cohort. All six finalized models were then evaluated without modification in the same independent test cohort. We replaced the principal tables and figures, corrected the regression results, added paired DeLong comparisons with Holm correction, and moderated the clinical claims. Changes are highlighted in the marked manuscript.

**Response to Journal Requirements**

**J.1 Reviewer or editor comment**

*Please ensure that your manuscript meets PLOS ONE's style requirements, including those for file naming.*

**Response:** We have prepared the marked revised manuscript and the clean manuscript as separate files and have reviewed the manuscript for terminology, typographical consistency, and table and figure presentation. The files will be uploaded using the submission labels specified by PLOS ONE.

**Changes in the revised manuscript:** The revision package contains a marked manuscript and a clean manuscript as separate submission files.

**J.2 Reviewer or editor comment**

*Please review the PLOS ONE guidelines on sharing author-generated code and ensure that the code is shared in a way that facilitates reproducibility and reuse.*

**Response:** We agree and have made the author-generated analysis code publicly accessible in the following GitHub repository: https://github.com/284496/Nsclc-lnm-petct. The repository contains the code used for the stratified cohort split, training-only logistic-regression screening, nested cross-validation, evaluation of the six finalized classifiers, paired DeLong comparisons, and SHAP analysis. No patient-level clinical data are included in the public repository.

**Changes in the revised manuscript:** A Code Availability statement should cite the public GitHub repository.

**J.3 Reviewer or editor comment**

*Please state what role the funders took in the study. If the funders had no role, please use the statement provided by the journal.*

**Response:** The Role of Funder statement in the cover letter and submission record will be revised to read: 'The funders had no role in study design, data collection and analysis, decision to publish, or preparation of the manuscript.'

**Changes in the revised manuscript:** This statement belongs in the cover letter and online submission record, as requested by the journal.

**J.4 Reviewer or editor comment**

*Please provide a general contact email address for data requests, or a website where readers can obtain access to data in perpetuity.*

**Response:** We corrected the institution responsible for data governance to Hunan Cancer Hospital. Because the data contain potentially identifying patient information, de-identified data may be made available to qualified researchers upon reasonable request and subject to approval by the Medical Ethics Committee of Hunan Cancer Hospital.

**Changes in the revised manuscript:** The Data Availability Statement now identifies Hunan Cancer Hospital as the responsible institution.

**J.5 Reviewer or editor comment**

*Reviewer-recommended publications should be evaluated for relevance; citation is not required unless indicated by the editor.*

**Response:** No reviewer requested citation of a specific publication. We therefore did not add references solely in response to a presumed citation request.

**Changes in the revised manuscript:** No change was required for this item.

**Response to the Academic Editor**

**E.1 Reviewer or editor comment**

*Feature selection appears to have been performed before the train-test split, while the test set was subsequently used to compare algorithms and select the final model. The analysis should be repeated using a leakage-free pipeline.*

**Response:** We agree. We repeated the analysis from the original patient-level data. First, we performed a random 70:30 patient-level split stratified by pathological lymph-node status, yielding a training cohort of 178 patients (40 LNM-positive and 138 LNM-negative) and an independent test cohort of 77 patients (18 LNM-positive and 59 LNM-negative). Univariable and multivariable logistic regression was then performed exclusively in the training cohort. Oversampling with SMOTEN, scaling where required by the algorithm, hyperparameter search, model ranking, and cutoff selection were confined to the training process. Model development used repeated stratified five-fold outer cross-validation with three repeats and four-fold inner hyperparameter search. XGBoost was designated the primary model because it achieved the highest training out-of-fold AUC. All six finalized models were subsequently applied without modification to the independent test cohort. Test-cohort comparisons did not alter the training-based selection.

**Changes in the revised manuscript:** Abstract; Materials and methods, Data Processing and Model Construction and Evaluation; Results, Model Performance; Discussion; Conclusion; Tables 1-3; Figures 1-4.

**E.2 Reviewer or editor comment**

*Please clarify the pathological definition of lymph-node status.*

**Response:** We clarified that all included patients underwent systematic lymph-node dissection and that no patients assessed solely by lymph-node sampling or biopsy were included. Postoperative histopathological examination of the systematically dissected lymph nodes served as the reference standard. Pathological N1 and N2 metastases were combined into the LNM-positive endpoint; patients without pathological N1 or N2 metastasis were classified as LNM-negative.

**Changes in the revised manuscript:** Materials and methods, Patients and imaging-variable definitions.

**E.3 Reviewer or editor comment**

*Please provide complete training/test cohort information, verify the reported statistical inconsistencies, moderate claims regarding clinical utility, and reconcile the Data Availability Statement with the study institution.*

**Response:** We replaced Table 1 with complete overall, training, and independent test cohort characteristics. We repeated the logistic-regression analysis in the training cohort and corrected Table 2. We replaced Table 3 with training out-of-fold and independent test performance for all six finalized algorithms. In the independent test cohort, AUCs ranged from 0.718 to 0.747, and none of the 15 paired DeLong comparisons was statistically significant after Holm correction. The primary XGBoost model achieved an AUC of 0.747 (95% CI, 0.631-0.846), sensitivity of 0.778, specificity of 0.644, PPV of 0.400, NPV of 0.905, accuracy of 0.675, and Brier score of 0.198. We now characterize this performance as moderate and present the model as an adjunctive risk-stratification tool requiring external validation. The Data Availability Statement now identifies Hunan Cancer Hospital rather than the Second Affiliated Hospital of Harbin Medical University.

**Changes in the revised manuscript:** Abstract; Results; Discussion; Conclusion; Tables 1-3; Data Availability Statement.

**Response to Reviewer 1**

**R1.1 Reviewer or editor comment**

*Feature selection was performed before the train-test split, and the same test set was then used to compare algorithms and select XGBoost. Thus, the independent test set participated in feature and model selection.*

**Response:** We agree that the original workflow did not preserve test-set independence. The analysis was rebuilt so that the stratified patient-level split preceded all model-development procedures. Feature screening, preprocessing, SMOTEN, hyperparameter optimization, model ranking, and cutoff determination used the training cohort only. XGBoost was selected solely from training out-of-fold AUC. The same independent test cohort was then used once to evaluate all six finalized models; these test results were not used for further tuning or selection.

**Changes in the revised manuscript:** Materials and methods, Data Processing and Model Construction and Evaluation; Results; Tables 1-3.

**R1.2 Reviewer or editor comment**

*The pathological definition of lymph-node status is unclear, particularly for LN-negative and biopsy-only cases.*

**Response:** We clarified the reference standard. All 255 patients underwent systematic lymph-node dissection, and no cases based solely on lymph-node sampling or biopsy were included. Postoperative histopathology of the dissected lymph nodes determined the endpoint. Pathological N1 and N2 disease was coded as LNM-positive, whereas the absence of pathological N1 or N2 disease was coded as LNM-negative.

**Changes in the revised manuscript:** Materials and methods, Patients and imaging-variable definitions.

**R1.3 Reviewer or editor comment**

*Several statistical results are difficult to reconcile, including the CTR findings and the identical odds ratio reported for CTR and tumor margin.*

**Response:** We repeated the analyses using the training cohort only. Because the sparse original nodule categories produced very few positive events outside the solid category, nodule status was represented as solid versus non-solid for regression and modeling. The corrected univariable OR for solid versus non-solid nodules was 17.65 (95% CI, 2.35-132.72; P=0.005), and the adjusted OR was 9.26 (95% CI, 1.11-77.10; P=0.039). Tumor margin was not significant in univariable analysis (OR, 1.27; 95% CI, 0.59-2.71; P=0.541) and was not entered into the multivariable model. The previously duplicated OR has been removed.

**Changes in the revised manuscript:** Materials and methods, Data Processing; Results, Model Performance; Table 2.

**R1.4 Reviewer or editor comment**

*The final performance is only moderate, with no convincing comparison with routine PET/CT assessment.*

**Response:** We agree that the corrected performance is moderate rather than favorable. The independent test AUC was 0.747 and the PPV was 0.400. The retrospective dataset did not contain a standardized radiologist nodal assessment, clinical N category, or PET/CT node-negative indicator, so a valid head-to-head comparison with routine nodal staging or a cN0/PET-negative subgroup analysis could not be reconstructed. We therefore removed claims of superiority or immediate clinical utility and now describe the model as an adjunctive risk-stratification tool that requires external and clinically targeted validation.

**Changes in the revised manuscript:** Abstract; Results, Model Performance; Discussion; Conclusion.

**R1.5 Reviewer or editor comment**

*SHAP plots cannot compensate for information leakage, uncertain labels, and questionable statistics.*

**Response:** We agree. SHAP was recalculated only after completion of the leakage-free training workflow and finalization of the primary XGBoost model. The revised manuscript uses SHAP only to describe how the finalized model distributes feature contributions. We do not treat SHAP as evidence of causal effects, independent validation, or clinical utility.

**Changes in the revised manuscript:** Materials and methods, Model Interpretation; Results, Interpreting the Model; Discussion; Figures 3 and 4.

**Response to Reviewer 2**

**R2.1 Reviewer or editor comment**

*Please clarify the intended clinical setting. The predictors may represent a recognizable high-risk phenotype. If possible, evaluate performance in clinical N0 or PET/CT node-negative patients.*

**Response:** We agree that this is an important clinical distinction. In the revised SHAP analysis, solid nodule status had the greatest global contribution, supporting the interpretation that the model partly captures a recognizable high-risk phenotype. We therefore narrowed the intended role to adjunctive preoperative risk stratification and explicitly state that the model should not replace conventional nodal staging. A separate cN0 or PET/CT node-negative analysis could not be performed because standardized clinical N category and node-level PET/CT assessment were not retained in the retrospective analysis dataset. Whether the model can identify occult nodal metastasis in this subgroup remains uncertain and requires targeted validation.

**Changes in the revised manuscript:** Abstract; Results, Interpreting the Model; Discussion; Conclusion.

**R2.2 Reviewer or editor comment**

*The performance appears moderate. Explain why XGBoost was considered the best model when the decision tree showed higher test-set metrics, and avoid overstating clinical utility.*

**Response:** We agree. The original test-based model selection was not valid. In the corrected workflow, XGBoost was designated the primary model before test evaluation because it had the highest training out-of-fold AUC (0.735; 95% CI, 0.653-0.813). For transparent secondary comparison, all six finalized models were evaluated in the same independent test cohort. The test AUCs were 0.747 for XGBoost, 0.747 for gradient boosting, 0.739 for extra trees, 0.739 for decision tree, 0.730 for SVM, and 0.718 for ANN. None of the 15 paired DeLong comparisons remained significant after Holm correction. We therefore describe XGBoost as the training-selected primary model, not as statistically superior to the other classifiers, and have moderated all clinical claims.

**Changes in the revised manuscript:** Abstract; Materials and methods, Model Construction and Evaluation; Results; Discussion; Conclusion; Table 3.

**R2.3 Reviewer or editor comment**

*Please clarify the order of splitting, feature selection, normalization, SMOTE, model training, hyperparameter tuning, and cross-validation. Provide exact positive and negative cohort counts and add calibration analysis.*

**Response:** The revised Methods now state the complete order. First, the cohort was randomly split at the patient level in a stratified 70:30 ratio. Second, univariable and multivariable screening was performed only in the training cohort. Third, model development used repeated stratified five-fold outer cross-validation with three repeats and four-fold inner randomized hyperparameter search. SMOTEN was applied only within cross-validation training folds because the four final predictors were binary. Scaling was included only where appropriate for the algorithm and was fitted within the training process. The operating threshold was derived from training out-of-fold predictions using the Youden index and fixed before independent test evaluation. The training cohort contained 40 LNM-positive and 138 LNM-negative patients; the test cohort contained 18 LNM-positive and 59 LNM-negative patients. We added Brier scores and calibration analysis, alongside ROC, precision-recall, and decision-curve analyses.

**Changes in the revised manuscript:** Abstract; Materials and methods, Data Processing and Model Construction and Evaluation; Results; Tables 1 and 3; Figures 1 and 2.

**R2.4 Reviewer or editor comment**

*Please provide details of pathological determination of LNM, including systematic dissection versus sampling and whether N1 and N2 metastases were combined.*

**Response:** All included patients underwent systematic lymph-node dissection. No patients assessed solely by lymph-node sampling or biopsy were included. Postoperative histopathology of the systematically dissected lymph nodes served as the reference standard. Pathological N1 and N2 metastases were combined into the LNM-positive endpoint, whereas patients without pathological N1 or N2 disease were classified as LNM-negative.

**Changes in the revised manuscript:** Materials and methods, Patients and imaging-variable definitions.

**R2.5 Reviewer or editor comment**

*The Data Availability Statement refers to a different institution from the Methods. Please correct this and clearly describe the procedure for requesting de-identified data.*

**Response:** We corrected the responsible institution to Hunan Cancer Hospital. Because the dataset contains potentially identifying clinical information, it is not posted publicly. De-identified data may be made available to qualified researchers upon reasonable request and subject to approval by the Medical Ethics Committee of Hunan Cancer Hospital.

**Changes in the revised manuscript:** Data Availability Statement.

**R2.6a Reviewer or editor comment**

*Please clarify the handling of missing data.*

**Response:** No missing values were present among the variables included in the analysis; therefore, no imputation was required in practice and no complete-case exclusion was performed.

**Changes in the revised manuscript:** This clarification should be added to Materials and methods, Data Processing.

**R2.6b Reviewer or editor comment**

*Please clarify the definition and measurement of CT value.*

**Response:** We clarified that CT attenuation was measured on the maximum axial cross-section of the primary tumor. The region of interest was placed to avoid visible necrosis, vessels, and calcification, and the mean attenuation value was recorded in Hounsfield units.

**Changes in the revised manuscript:** Materials and methods, imaging-variable definitions.

**R2.6c Reviewer or editor comment**

*Please clarify the rationale for the CEA and SCC cutoffs.*

**Response:** CEA and SCC antigen were already recorded in the source dataset as binary variables (normal or elevated) according to the institutional laboratory reference range in effect at the time of testing. Values flagged above the corresponding upper limit of normal in the laboratory report were classified as elevated. No data-driven cutoff was selected or optimized against LNM outcome, and the analysis dataset did not retain the original continuous concentrations; therefore, we do not report unsupported fixed numerical cutoffs.

**Changes in the revised manuscript:** This clarification should be added to Materials and methods, Patients or Data Processing.

**R2.6d Reviewer or editor comment**

*Please clarify whether the training-test split was random or stratified.*

**Response:** The 70:30 patient-level split was random and stratified by pathological lymph-node status, using a fixed random seed of 42. This produced comparable LNM prevalence in the training cohort (22.5%) and test cohort (23.4%).

**Changes in the revised manuscript:** Materials and methods, Data Processing; Results, Patient Characteristics; Table 1.

**R2.6e Reviewer or editor comment**

*Some minor language and typographical corrections are needed.*

**Response:** We revised the manuscript for terminology, grammar, spacing, punctuation, and consistency of abbreviations and model names.

We again thank the Academic Editor and Reviewers for identifying the methodological and reporting issues in the original submission. We believe that the leakage-free reanalysis, corrected statistical reporting, clarified pathological reference standard, and more cautious interpretation substantially improve the rigor and transparency of the manuscript.

Sincerely,
The Authors
