# Supplementary material for: Predicting lymph node metastasis in non-small cell lung cancer using clinical and PET/CT-derived morphological features: An interpretable machine-learning study
Source: PLoS One. 2026 Oct 15;21(10):e0361043. doi: 10.1371/journal.pone.0361043 (PMC13671544; doi:10.1371/journal.pone.0361043)
Supplement: Supplementary file 2 [file pone.0361043.s004.docx]

**Response to Reviewers**

**Manuscript ID:** PONE-D-26-39894R1

**Title:** Predicting lymph node metastasis in non-small cell lung cancer using clinical and PET/CT-derived morphological features: an interpretable machine-learning study

Dear Academic Editor and Reviewers,

We thank you for the careful evaluation of our revised manuscript and for the focused suggestions for minor revision. We have addressed every comment. The principal changes include clarification that the final imaging predictors are morphological rather than quantitative PET metabolic parameters; addition of a conventional multivariable logistic regression reference model using the same four predictors; reporting of calibration intercepts and slopes; more cautious interpretation of the negative predictive value; clearer description of the internal held-out test cohort and single-center limitations; and inclusion of recent relevant studies. We also added the requested information on missing data and the coding of CEA and SCC antigen. All changes are shown in red bold text in the marked manuscript.

**Response to Reviewer 1**

**Comment 1**

*The final model contains only four binary predictors: central tumor location, elevated CEA, elevated SCC antigen, and solid nodule status. None of the quantitative PET parameters, including SUVmax, SUVmean, MTV, or TLG, remained in the final model. The title and Discussion should therefore avoid giving the impression that PET metabolic information provides independent predictive value. Please clarify that the final imaging contribution is mainly based on morphological features obtained from the PET/CT examination.*

**Response:** We agree. We revised the title and clarified in the Abstract, Results, Discussion, and Conclusion that SUVmax, SUVmean, MTV, and TLG were evaluated but were not retained after multivariable adjustment. The final imaging contribution therefore comes primarily from CT-based morphological characteristics obtained during the PET/CT examination, especially tumor location and solid nodule status, rather than from independent quantitative PET metabolic information.

**Changes in the manuscript: Title; Abstract; Results, Model Performance; Discussion, first paragraph; and Conclusion. The revised title now refers to ‘clinical and PET/CT-derived morphological features.’**

**Comment 2**

*Since the final model contains only four binary variables, I suggest adding a conventional multivariable logistic regression model using exactly the same predictors as a simple reference model. Comparing its AUC, calibration, and Brier score with XGBoost would help determine whether the additional machine-learning complexity provides meaningful benefit.*

**Response:** We performed the requested reference analysis using an unpenalized multivariable logistic regression model containing exactly the same four fixed predictors, without further feature selection or oversampling. Training performance was estimated by repeated stratified five-fold cross-validation with three repeats. The final logistic model was then fitted to the complete original training cohort and applied without modification to the internal held-out test cohort. Logistic regression achieved a test AUC of 0.733 (95% CI, 0.612–0.841), compared with 0.747 (95% CI, 0.631–0.846) for XGBoost. The paired DeLong comparison was not significant (P=0.301). The test Brier score was 0.152 for logistic regression and 0.198 for XGBoost. These findings provide no clear evidence that the additional complexity of XGBoost improved performance in this dataset.

**Changes in the manuscript: Methods, Model Development and Statistical Analysis; Results, Model Performance; Table 3; Discussion, first paragraph; Conclusion; and S1 Fig.**

**Comment 3**

*The calibration analysis could be reported more clearly. In addition to the Brier score and calibration plot, please consider providing the calibration intercept and slope. The high NPV should also be interpreted in the context of the relatively low LNM prevalence and should not be taken as evidence that the model can safely exclude nodal metastasis in clinically node-negative patients.*

**Response:** We added calibration intercepts and slopes with bootstrap 95% confidence intervals. In the internal held-out test cohort, the XGBoost calibration intercept was −1.140 (95% CI, −1.946 to −0.628) and the slope was 1.031 (95% CI, 0.568–1.930). For logistic regression, the intercept was 0.207 (95% CI, −0.649 to 1.278) and the slope was 1.095 (95% CI, 0.612–2.109). We also added S1 Fig to compare calibration graphically. In the Discussion, we now state that the XGBoost NPV of 0.905 is influenced by the test-cohort LNM prevalence of 23.4% and must not be interpreted as evidence that the model can safely exclude nodal metastasis in clinically node-negative patients.

**Changes in the manuscript: Methods, Model Development and Statistical Analysis; Results, Model Performance; Discussion, first paragraph; and S1 Fig.**

**Comment 4**

*Please continue to distinguish an internal held-out test cohort from true external validation. The current results are encouraging but remain single-center. In addition, the wide confidence interval for solid nodule status suggests some instability of this predictor, and this should be acknowledged when discussing SHAP importance and clinical interpretation.*

**Response:** We revised the manuscript to use the term ‘internal held-out test cohort’ consistently and explicitly state that the study was retrospective and single-center and that no external validation was performed. We also added a caution that the wide confidence interval for solid nodule status (OR, 9.26; 95% CI, 1.11–77.10) indicates uncertainty in the stability of this predictor. Its leading SHAP ranking is therefore interpreted as a contribution within the fitted model, not as definitive evidence of a stable or causal clinical effect.

**Changes in the manuscript: Abstract; Methods, Data Processing and Model Development; Results, Model Performance and Interpreting the Model; Discussion, limitations; and Conclusion.**

**Comment 5**

*The Discussion would benefit from a more up-to-date comparison with recent multicenter imaging studies of lymph-node metastasis. I suggest that the authors consider recent CT studies such as DOI: 10.1016/j.acra.2025.06.001 and DOI: 10.21147/j.issn.1000-9604.2025.01.02. Related methodological work may also be useful, particularly DOI: 10.1186/s12916-026-04865-1 and DOI: 10.1007/s00259-025-07090-9.*

**Response:** We reviewed all four suggested articles and found them relevant. The two recent multicenter CT studies are now discussed as direct examples of imaging-based LNM prediction with external validation. The two multimodal PET/CT studies in other malignancies are cited as related methodological work on explainable imaging-clinical integration. We have avoided direct numerical comparison because the populations, outcomes, and feature sets differ from those in our study.

**Changes in the manuscript: Discussion, second paragraph; new References 18–20.**

**Response to Reviewer 2**

**Comment 1**

*The response states that there were no missing values and that neither imputation nor complete-case exclusion was required. Please add this information clearly to the Methods section.*

**Response:** We added this information explicitly. No values were missing for the variables included in the analyses; therefore, neither data imputation nor complete-case exclusion was required.

**Changes in the manuscript: Methods, Data Processing.**

**Comment 2**

*The response also explains that CEA and SCC were recorded as normal or elevated according to the institutional laboratory reference ranges, and that the original continuous values were not available. This should also be stated in the manuscript.*

**Response:** We added this information to the Methods. CEA and SCC antigen were recorded as normal or elevated according to the institutional laboratory reference ranges applicable at the time of measurement, and the original continuous concentrations were not available for this analysis.

**Changes in the manuscript: Methods, Patients and variable collection.**

**Comment 3**

*In the Discussion, please make it clear that the added value of this model over conventional clinical and PET/CT nodal staging cannot be assessed from the present dataset, since standardized clinical N category and node-level PET/CT assessment were not available.*

**Response:** We agree and have added this limitation explicitly. Because standardized clinical N category and node-level PET/CT assessments were unavailable, the incremental value of the model beyond conventional clinical and PET/CT nodal staging could not be evaluated. The manuscript also avoids claiming that the model can replace conventional staging or safely exclude metastasis in clinically node-negative patients.

**Changes in the manuscript: Discussion, limitations; Conclusion.**

We again thank the Academic Editor and Reviewers for their constructive comments, which helped us improve the clarity and balance of the manuscript.

Sincerely,

The Authors
