# Supplementary material for: Predicting lymph node metastasis in non-small cell lung cancer using clinical and PET/CT-derived morphological features: An interpretable machine-learning study
Source: PLoS One. 2026 Oct 15;21(10):e0361043. doi: 10.1371/journal.pone.0361043 (PMC13671544; doi:10.1371/journal.pone.0361043)
Supplement: Peer review history

# Peer review: Decision Letter 0

**DOI:** 10.1371/journal.pone.0361043.r001

**Authors:** Chong Jiang

30 Aug 2026

Dear Dr. Xu,

Thank you for submitting your manuscript to PLOS One. After careful consideration, we feel that it has merit but does not fully meet PLOS One’s publication criteria as it currently stands. Therefore, we invite you to submit a revised version of the manuscript that addresses the points raised during the review process.

Please submit your revised manuscript by Oct 29 2026 11:59PM. If you will need more time than this to complete your revisions, please reply to this message or contact the journal office at plosone@plos.org. When you're ready to submit your revision, log on to https://www.editorialmanager.com/pone/ and select the 'Submissions Needing Revision' folder to locate your manuscript file.

- A letter that responds to each point raised by the academic editor and reviewer(s). You should upload this letter as a separate file labeled 'Response to Reviewers'.
- A marked-up copy of your manuscript that highlights changes made to the original version. You should upload this as a separate file labeled 'Revised Manuscript with Track Changes'.
- An unmarked version of your revised paper without tracked changes. You should upload this as a separate file labeled 'Manuscript'.

If you would like to make changes to your financial disclosure, please include your updated statement in your cover letter. Guidelines for resubmitting your figure files are available below the reviewer comments at the end of this letter.

If applicable, we recommend that you deposit your laboratory protocols in protocols.io to enhance the reproducibility of your results. Protocols.io assigns your protocol its own identifier (DOI) so that it can be cited independently in the future. For instructions see: https://journals.plos.org/plosone/s/submission-guidelines#loc-laboratory-protocols. Additionally, PLOS One offers an option for publishing peer-reviewed Lab Protocol articles, which describe protocols hosted on protocols.io. Read more information on sharing protocols at https://plos.org/protocols?utm_medium=editorial-email&utm_source=authorletters&utm_campaign=protocols.

As the corresponding author, your ORCID iD is verified in the submission system and will appear in the published article. PLOS supports the use of ORCID, and we encourage all coauthors to register for an ORCID iD and use it as well. Please encourage your coauthors to verify their ORCID iD within the submission system before final acceptance, as unverified ORCID iDs will not appear in the published article. *Only*  the individual author can complete the verification step; PLOS staff *cannot*  verify ORCID iDs on behalf of authors.

We look forward to receiving your revised manuscript.

Kind regards,

Chong Jiang

Academic Editor

PLOS One

Journal Requirements:

When submitting your revision, we need you to address these additional requirements.

1. Please ensure that your manuscript meets PLOS ONE's style requirements, including those for file naming. The PLOS ONE style templates can be found at

https://journals.plos.org/plosone/s/file?id=wjVg/PLOSOne_formatting_sample_main_body.pdf and

https://journals.plos.org/plosone/s/file?id=ba62/PLOSOne_formatting_sample_title_authors_affiliations.pdf

2. Please note that PLOS One has specific guidelines on code sharing for submissions in which author-generated code underpins the findings in the manuscript. In these cases, we expect all author-generated code to be made available without restrictions upon publication of the work. Please review our guidelines at https://journals.plos.org/plosone/s/materials-and-software-sharing#loc-sharing-code and ensure that your code is shared in a way that follows best practice and facilitates reproducibility and reuse.

3. Thank you for stating the following financial disclosure:

“This work was supported by the Hunan Provincial Natural Science Foundation and Health Joint Fund 2026JJ81742, the Hunan Provincial Health Commission Health Appropriate Technology Promotion Project 202319015975, the High-Level Talent Support Program of Hunan Cancer Hospital 20250731-1009; 20250731-1016, China.”

Please state what role the funders took in the study.  If the funders had no role, please state: "The funders had no role in study design, data collection and analysis, decision to publish, or preparation of the manuscript."

If this statement is not correct you must amend it as needed.

Please include this amended Role of Funder statement in your cover letter; we will change the online submission form on your behalf.

4. You have indicated that data is available from 359368584@qq.com.  Please can we ask you to provide us with a general contact email address for the data requests, so readers can request access in perpetuity. If a general email is not available please provide a link to a website where readers can obtain access to data.

5. If the reviewer comments include a recommendation to cite specific previously published works, please review and evaluate these publications to determine whether they are relevant and should be cited. There is no requirement to cite these works unless the editor has indicated otherwise.

Additional Editor Comments:

Dear Authors,

Thank you for submitting your manuscript to PLOS ONE.

After careful consideration of the manuscript and the reviewers’ comments, I have decided that a major revision is required.

The principal concern is the model-development and validation workflow. Feature selection appears to have been performed before the train-test split, while the test set was subsequently used to compare algorithms and select the final model. This compromises the independence of the test set and requires the analysis to be repeated using a leakage-free pipeline, with all feature selection, preprocessing, hyperparameter tuning, and model selection confined to the training data.

Please also clarify the pathological definition of lymph-node status, provide complete training/test cohort information, verify the reported statistical inconsistencies, and moderate claims regarding clinical utility given the current performance. The Data Availability Statement should also be reconciled with the study institution.

Please address all reviewer comments in a detailed point-by-point response. The revised conclusions should be based on the corrected analyses.

Sincerely,

Academic Editor

PLOS ONE

[Note: HTML markup is below. Please do not edit.]

Reviewers' comments:

Reviewer's Responses to Questions

**Comments to the Author**

1. Is the manuscript technically sound, and do the data support the conclusions?

Reviewer #1: No

Reviewer #2: Yes

**********

2. Has the statistical analysis been performed appropriately and rigorously?

Reviewer #1: No

Reviewer #2: Yes

**********

3. Have the authors made all data underlying the findings in their manuscript fully available?

The PLOS Data policy

Reviewer #1: Yes

Reviewer #2: No

**********

4. Is the manuscript presented in an intelligible fashion and written in standard English?

Reviewer #1: No

Reviewer #2: Yes

**********

Reviewer #1: The topic is relevant, but the manuscript gives the impression of technical sophistication without the methodological rigor to support it. Feature selection was performed before the train-test split, and the same test set was then used to compare algorithms and select XGBoost. Thus, the “independent” test set participates in both feature selection and model selection—a basic validation error that raises concerns about the authors’ understanding of prediction-model methodology. The pathological definition of LN status is also unclear, particularly for LN-negative and biopsy-only cases. In addition, several statistical results are difficult to reconcile, including the CTR findings and the identical OR reported for CTR and tumor margin, which makes me question whether the authors adequately checked their own analysis. The final performance is only moderate, with an AUC of 0.801, specificity of 0.661, and precision of 0.394, and no convincing comparison with routine PET/CT assessment is provided. SHAP plots may make the model look more sophisticated, but they cannot rescue information leakage, uncertain labels, and questionable statistics. The analysis would need to be rebuilt from the ground up. I therefore recommend rejection.

Reviewer #2: This is an interesting study evaluating a machine learning model for prediction of lymph node metastasis (LNM) in NSCLC using clinical and PET/CT parameters. The results are generally understandable, but I have several concerns that should be addressed before publication.

Major comments

1. Clinical relevance of the prediction model

My main concern is what kind of LNM this model is actually predicting. Only 58 of 255 patients had LNM. In the LNM-positive group, central tumors and squamous cell carcinoma were more frequent, and 57 of 58 LNM-positive tumors were solid nodules. The final predictors, including tumor location, SUVmean, CT value, and SCC antigen, may therefore represent a relatively typical high-risk phenotype, such as a central, solid, metabolically active squamous cell carcinoma.

Although the model may correctly identify this phenotype, these patients may already be considered at high risk of nodal involvement in routine clinical practice. The authors should clarify the intended clinical setting of the model. If possible, it would be more clinically informative to evaluate its performance in patients with less obvious nodal disease, particularly clinical N0 or PET/CT node-negative patients.

2. Predictive performance

The test-set performance of XGBoost (AUC 0.801, sensitivity 0.722, specificity 0.661) appears moderate rather than clearly “favorable.” In addition, the decision tree showed higher accuracy, sensitivity, specificity, precision, and F1-score than XGBoost in the test set. The authors should explain more carefully why XGBoost was considered the best model and avoid overstating its clinical utility.

3. Model development and validation

The Methods appear to indicate that univariate and multivariable analyses for feature selection were performed before the train-test split. Please clarify the exact order of data splitting, feature selection, normalization, SMOTE, and model training. All model-development procedures should be performed using the training data only. The authors should also provide more details regarding hyperparameter tuning and cross-validation.

The number of LNM-positive patients is relatively small, particularly in the test set. Exact numbers of positive and negative cases in the training and test sets should be provided. Calibration analysis would also strengthen the evaluation of the model.

4. Definition of LNM

Please provide more details regarding the pathological determination of LNM, including whether systematic lymph node dissection or sampling was performed and whether N1 and N2 metastases were combined into a single endpoint.

5. Data availability

There is an inconsistency regarding the institution responsible for data access. The Data Availability Statement refers to the Second Affiliated Hospital of Harbin Medical University, whereas the Methods identify the Ethics Committee of Hunan Cancer Hospital. This should be corrected, and the procedure for requesting de-identified data should be clearly described.

Minor comments

Please clarify the handling of missing data, the definition and measurement of CT value, the rationale for the CEA and SCC cutoffs, and whether the train-test split was random or stratified. Some minor language and typographical corrections are also needed.

Overall, the study has potential, but the clinical role of the model should be better defined. In particular, demonstrating predictive value beyond an already recognizable high-risk tumor phenotype would substantially strengthen the manuscript.

**********

what does this mean?). If published, this will include your full peer review and any attached files.

If you choose “no”, your identity will remain anonymous but your review may still be made public.

**Do you want your identity to be public for this peer review?**  For information about this choice, including consent withdrawal, please see our Privacy Policy

Reviewer #1: No

Reviewer #2: No

**********

[NOTE: If reviewer comments were submitted as an attachment file, they will be attached to this email and accessible via the submission site. Please log into your account, locate the manuscript record, and check for the action link "View Attachments". If this link does not appear, there are no attachment files.]

To ensure your figures meet our technical requirements, please review our figure guidelines: https://journals.plos.org/plosone/s/figures

You may also use PLOS’s free figure tool, NAAS, to help you prepare publication quality figures: https://journals.plos.org/plosone/s/figures#loc-tools-for-figure-preparation.

NAAS will assess whether your figures meet our technical requirements by comparing each figure against our figure specifications.

# Author comment: Author response to Decision Letter 1

**DOI:** 10.1371/journal.pone.0361043.r002

9 Sep 2026

Response to Reviewers

Manuscript ID: PONE-D-26-39894

Title: Predicting lymph node metastasis in non-small cell lung cancer combining clinical and PET/CT features with machine learning: an interpretable SHAP-based study

Dear Academic Editor and Reviewers,

We thank the Academic Editor and Reviewers for their careful and constructive evaluation of our manuscript. We rebuilt the analysis from the original patient-level data using a leakage-free workflow. The patient-level training-test split was fixed before feature screening, and all feature selection, preprocessing, oversampling, hyperparameter tuning, algorithm ranking, and cutoff selection were confined to the training cohort. All six finalized models were then evaluated without modification in the same independent test cohort. We replaced the principal tables and figures, corrected the regression results, added paired DeLong comparisons with Holm correction, and moderated the clinical claims. Changes are highlighted in the marked manuscript.

Response to Journal Requirements

J.1 Reviewer or editor comment

Please ensure that your manuscript meets PLOS ONE's style requirements, including those for file naming.

Response: We have prepared the marked revised manuscript and the clean manuscript as separate files and have reviewed the manuscript for terminology, typographical consistency, and table and figure presentation. The files will be uploaded using the submission labels specified by PLOS ONE.

Changes in the revised manuscript: The revision package contains a marked manuscript and a clean manuscript as separate submission files.

J.2 Reviewer or editor comment

Please review the PLOS ONE guidelines on sharing author-generated code and ensure that the code is shared in a way that facilitates reproducibility and reuse.

Response: We agree and have made the author-generated analysis code publicly accessible in the following GitHub repository: https://github.com/284496/Nsclc-lnm-petct. The repository contains the code used for the stratified cohort split, training-only logistic-regression screening, nested cross-validation, evaluation of the six finalized classifiers, paired DeLong comparisons, and SHAP analysis. No patient-level clinical data are included in the public repository.

Changes in the revised manuscript: A Code Availability statement should cite the public GitHub repository.

J.3 Reviewer or editor comment

Please state what role the funders took in the study. If the funders had no role, please use the statement provided by the journal.

Response: The Role of Funder statement in the cover letter and submission record will be revised to read: 'The funders had no role in study design, data collection and analysis, decision to publish, or preparation of the manuscript.'

Changes in the revised manuscript: This statement belongs in the cover letter and online submission record, as requested by the journal.

J.4 Reviewer or editor comment

Please provide a general contact email address for data requests, or a website where readers can obtain access to data in perpetuity.

Response: We corrected the institution responsible for data governance to Hunan Cancer Hospital. Because the data contain potentially identifying patient information, de-identified data may be made available to qualified researchers upon reasonable request and subject to approval by the Medical Ethics Committee of Hunan Cancer Hospital.

Changes in the revised manuscript: The Data Availability Statement now identifies Hunan Cancer Hospital as the responsible institution.

J.5 Reviewer or editor comment

Reviewer-recommended publications should be evaluated for relevance; citation is not required unless indicated by the editor.

Response: No reviewer requested citation of a specific publication. We therefore did not add references solely in response to a presumed citation request.

Changes in the revised manuscript: No change was required for this item.

Response to the Academic Editor

E.1 Reviewer or editor comment

Feature selection appears to have been performed before the train-test split, while the test set was subsequently used to compare algorithms and select the final model. The analysis should be repeated using a leakage-free pipeline.

Response: We agree. We repeated the analysis from the original patient-level data. First, we performed a random 70:30 patient-level split stratified by pathological lymph-node status, yielding a training cohort of 178 patients (40 LNM-positive and 138 LNM-negative) and an independent test cohort of 77 patients (18 LNM-positive and 59 LNM-negative). Univariable and multivariable logistic regression was then performed exclusively in the training cohort. Oversampling with SMOTEN, scaling where required by the algorithm, hyperparameter search, model ranking, and cutoff selection were confined to the training process. Model development used repeated stratified five-fold outer cross-validation with three repeats and four-fold inner hyperparameter search. XGBoost was designated the primary model because it achieved the highest training out-of-fold AUC. All six finalized models were subsequently applied without modification to the independent test cohort. Test-cohort comparisons did not alter the training-based selection.

Changes in the revised manuscript: Abstract; Materials and methods, Data Processing and Model Construction and Evaluation; Results, Model Performance; Discussion; Conclusion; Tables 1-3; Figures 1-4.

E.2 Reviewer or editor comment

Please clarify the pathological definition of lymph-node status.

Response: We clarified that all included patients underwent systematic lymph-node dissection and that no patients assessed solely by lymph-node sampling or biopsy were included. Postoperative histopathological examination of the systematically dissected lymph nodes served as the reference standard. Pathological N1 and N2 metastases were combined into the LNM-positive endpoint; patients without pathological N1 or N2 metastasis were classified as LNM-negative.

Changes in the revised manuscript: Materials and methods, Patients and imaging-variable definitions.

E.3 Reviewer or editor comment

Please provide complete training/test cohort information, verify the reported statistical inconsistencies, moderate claims regarding clinical utility, and reconcile the Data Availability Statement with the study institution.

Response: We replaced Table 1 with complete overall, training, and independent test cohort characteristics. We repeated the logistic-regression analysis in the training cohort and corrected Table 2. We replaced Table 3 with training out-of-fold and independent test performance for all six finalized algorithms. In the independent test cohort, AUCs ranged from 0.718 to 0.747, and none of the 15 paired DeLong comparisons was statistically significant after Holm correction. The primary XGBoost model achieved an AUC of 0.747 (95% CI, 0.631-0.846), sensitivity of 0.778, specificity of 0.644, PPV of 0.400, NPV of 0.905, accuracy of 0.675, and Brier score of 0.198. We now characterize this performance as moderate and present the model as an adjunctive risk-stratification tool requiring external validation. The Data Availability Statement now identifies Hunan Cancer Hospital rather than the Second Affiliated Hospital of Harbin Medical University.

Changes in the revised manuscript: Abstract; Results; Discussion; Conclusion; Tables 1-3; Data Availability Statement.

Response to Reviewer 1

R1.1 Reviewer or editor comment

Feature selection was performed before the train-test split, and the same test set was then used to compare algorithms and select XGBoost. Thus, the independent test set participated in feature and model selection.

Response: We agree that the original workflow did not preserve test-set independence. The analysis was rebuilt so that the stratified patient-level split preceded all model-development procedures. Feature screening, preprocessing, SMOTEN, hyperparameter optimization, model ranking, and cutoff determination used the training cohort only. XGBoost was selected solely from training out-of-fold AUC. The same independent test cohort was then used once to evaluate all six finalized models; these test results were not used for further tuning or selection.

Changes in the revised manuscript: Materials and methods, Data Processing and Model Construction and Evaluation; Results; Tables 1-3.

R1.2 Reviewer or editor comment

The pathological definition of lymph-node status is unclear, particularly for LN-negative and biopsy-only cases.

Response: We clarified the reference standard. All 255 patients underwent systematic lymph-node dissection, and no cases based solely on lymph-node sampling or biopsy were included. Postoperative histopathology of the dissected lymph nodes determined the endpoint. Pathological N1 and N2 disease was coded as LNM-positive, whereas the absence of pathological N1 or N2 disease was coded as LNM-negative.

Changes in the revised manuscript: Materials and methods, Patients and imaging-variable definitions.

R1.3 Reviewer or editor comment

Several statistical results are difficult to reconcile, including the CTR findings and the identical odds ratio reported for CTR and tumor margin.

Response: We repeated the analyses using the training cohort only. Because the sparse original nodule categories produced very few positive events outside the solid category, nodule status was represented as solid versus non-solid for regression and modeling. The corrected univariable OR for solid versus non-solid nodules was 17.65 (95% CI, 2.35-132.72; P=0.005), and the adjusted OR was 9.26 (95% CI, 1.11-77.10; P=0.039). Tumor margin was not significant in univariable analysis (OR, 1.27; 95% CI, 0.59-2.71; P=0.541) and was not entered into the multivariable model. The previously duplicated OR has been removed.

Changes in the revised manuscript: Materials and methods, Data Processing; Results, Model Performance; Table 2.

R1.4 Reviewer or editor comment

The final performance is only moderate, with no convincing comparison with routine PET/CT assessment.

Response: We agree that the corrected performance is moderate rather than favorable. The independent test AUC was 0.747 and the PPV was 0.400. The retrospective dataset did not contain a standardized radiologist nodal assessment, clinical N category, or PET/CT node-negative indicator, so a valid head-to-head comparison with routine nodal staging or a cN0/PET-negative subgroup analysis could not be reconstructed. We therefore removed claims of superiority or immediate clinical utility and now describe the model as an adjunctive risk-stratification tool that requires external and clinically targeted validation.

Changes in the revised manuscript: Abstract; Results, Model Performance; Discussion; Conclusion.

R1.5 Reviewer or editor comment

SHAP plots cannot compensate for information leakage, uncertain labels, and questionable statistics.

Response: We agree. SHAP was recalculated only after completion of the leakage-free training workflow and finalization of the primary XGBoost model. The revised manuscript uses SHAP only to describe how the finalized model distributes feature contributions. We do not treat SHAP as evidence of causal effects, independent validation, or clinical utility.

Changes in the revised manuscript: Materials and methods, Model Interpretation; Results, Interpreting the Model; Discussion; Figures 3 and 4.

Response to Reviewer 2

R2.1 Reviewer or editor comment

Please clarify the intended clinical setting. The predictors may represent a recognizable high-risk phenotype. If possible, evaluate performance in clinical N0 or PET/CT node-negative patients.

Response: We agree that this is an important clinical distinction. In the revised SHAP analysis, solid nodule status had the greatest global contribution, supporting the interpretation that the model partly captures a recognizable high-risk phenotype. We therefore narrowed the intended role to adjunctive preoperative risk stratification and explicitly state that the model should not replace conventional nodal staging. A separate cN0 or PET/CT node-negative analysis could not be performed because standardized clinical N category and node-level PET/CT assessment were not retained in the retrospective analysis dataset. Whether the model can identify occult nodal metastasis in this subgroup remains uncertain and requires targeted validation.

Changes in the revised manuscript: Abstract; Results, Interpreting the Model; Discussion; Conclusion.

R2.2 Reviewer or editor comment

The performance appears moderate. Explain why XGBoost was considered the best model when the decision tree showed higher test-set metrics, and avoid overstating clinical utility.

Response: We agree. The original test-based model selection was not valid. In the corrected workflow, XGBoost was designated the primary model before test evaluation because it had the highest training out-of-fold AUC (0.735; 95% CI, 0.653-0.813). For transparent secondary comparison, all six finalized models were evaluated in the same independent test cohort. The test AUCs were 0.747 for XGBoost, 0.747 for gradient boosting, 0.739 for extra trees, 0.739 for decision tree, 0.730 for SVM, and 0.718 for ANN. None of the 15 paired DeLong comparisons remained significant after Holm correction. We therefore describe XGBoost as the training-selected primary model, not as statistically superior to the other classifiers, and have moderated all clinical claims.

Changes in the revised manuscript: Abstract; Materials and methods, Model Construction and Evaluation; Results; Discussion; Conclusion; Table 3.

R2.3 Reviewer or editor comment

Please clarify the order of splitting, feature selection, normalization, SMOTE, model training, hyperparameter tuning, and cross-validation. Provide exact positive and negative cohort counts and add calibration analysis.

Response: The revised Methods now state the complete order. First, the cohort was randomly split at the patient level in a stratified 70:30 ratio. Second, univariable and multivariable screening was performed only in the training cohort. Third, model development used repeated stratified five-fold outer cross-validation with three repeats and four-fold inner randomized hyperparameter search. SMOTEN was applied only within cross-validation training folds because the four final predictors were binary. Scaling was included only where appropriate for the algorithm and was fitted within the training process. The operating threshold was derived from training out-of-fold predictions using the Youden index and fixed before independent test evaluation. The training cohort contained 40 LNM-positive and 138 LNM-negative patients; the test cohort contained 18 LNM-positive and 59 LNM-negative patients. We added Brier scores and calibration analysis, alongside ROC, precision-recall, and decision-curve analyses.

Changes in the revised manuscript: Abstract; Materials and methods, Data Processing and Model Construction and Evaluation; Results; Tables 1 and 3; Figures 1 and 2.

R2.4 Reviewer or editor comment

Please provide details of pathological determination of LNM, including systematic dissection versus sampling and whether N1 and N2 metastases were combined.

Response: All included patients underwent systematic lymph-node dissection. No patients assessed solely by lymph-node sampling or biopsy were included. Postoperative histopathology of the systematically dissected lymph nodes served as the reference standard. Pathological N1 and N2 metastases were combined into the LNM-positive endpoint, whereas patients without pathological N1 or N2 disease were classified as LNM-negative.

Changes in the revised manuscript: Materials and methods, Patients and imaging-variable definitions.

R2.5 Reviewer or editor comment

The Data Availability Statement refers to a different institution from the Methods. Please correct this and clearly describe the procedure for requesting de-identified data.

Response: We corrected the responsible institution to Hunan Cancer Hospital. Because the dataset contains potentially identifying clinical information, it is not posted publicly. De-identified data may be made available to qualified researchers upon reasonable request and subject to approval by the Medical Ethics Committee of Hunan Cancer Hospital.

Changes in the revised manuscript: Data Availability Statement.

R2.6a Reviewer or editor comment

Please clarify the handling of missing data.

Response: No missing values were present among the variables included in the analysis; therefore, no imputation was required in practice and no complete-case exclusion was performed.

Changes in the revised manuscript: This clarification should be added to Materials and methods, Data Processing.

R2.6b Reviewer or editor comment

Please clarify the definition and measurement of CT value.

Response: We clarified that CT attenuation was measured on the maximum axial cross-section of the primary tumor. The region of interest was placed to avoid visible necrosis, vessels, and calcification, and the mean attenuation value was recorded in Hounsfield units.

Changes in the revised manuscript: Materials and methods, imaging-variable definitions.

R2.6c Reviewer or editor comment

Please clarify the rationale for the CEA and SCC cutoffs.

Response: CEA and SCC antigen were already recorded in the source dataset as binary variables (normal or elevated) according to the institutional laboratory reference range in effect at the time of testing. Values flagged above the corresponding upper limit of normal in the laboratory report were classified as elevated. No data-driven cutoff was selected or optimized against LNM outcome, and the analysis dataset did not retain the original continuous concentrations; therefore, we do not report unsupported fixed numerical cutoffs.

Changes in the revised manuscript: This clarification should be added to Materials and methods, Patients or Data Processing.

R2.6d Reviewer or editor comment

Please clarify whether the training-test split was random or stratified.

Response: The 70:30 patient-level split was random and stratified by pathological lymph-node status, using a fixed random seed of 42. This produced comparable LNM prevalence in the training cohort (22.5%) and test cohort (23.4%).

Changes in the revised manuscript: Materials and methods, Data Processing; Results, Patient Characteristics; Table 1.

R2.6e Reviewer or editor comment

Some minor language and typographical corrections are needed.

Response: We revised the manuscript for terminology, grammar, spacing, punctuation, and consistency of abbreviations and model names.

We again thank the Academic Editor and Reviewers for identifying the methodological and reporting issues in the original submission. We believe that the leakage-free reanalysis, corrected statistical reporting, clarified pathological reference standard, and more cautious interpretation substantially improve the rigor and transparency of the manuscript.

Sincerely,

The Authors

Attachment Submitted filename: Response to Reviewers_with locations.docx

# Peer review: Decision Letter 1

**DOI:** 10.1371/journal.pone.0361043.r003

**Authors:** Chong Jiang

15 Sep 2026

Dear Dr. Xu,

Thank you for submitting your manuscript to PLOS One. After careful consideration, we feel that it has merit but does not fully meet PLOS One’s publication criteria as it currently stands. Therefore, we invite you to submit a revised version of the manuscript that addresses the points raised during the review process.

Please submit your revised manuscript by Oct 30 2026 11:59PM. If you will need more time than this to complete your revisions, please reply to this message or contact the journal office at plosone@plos.org. When you're ready to submit your revision, log on to https://www.editorialmanager.com/pone/ and select the 'Submissions Needing Revision' folder to locate your manuscript file.

- A letter that responds to each point raised by the academic editor and reviewer(s). You should upload this letter as a separate file labeled 'Response to Reviewers'.
- A marked-up copy of your manuscript that highlights changes made to the original version. You should upload this as a separate file labeled 'Revised Manuscript with Track Changes'.
- An unmarked version of your revised paper without tracked changes. You should upload this as a separate file labeled 'Manuscript'.

If you would like to make changes to your financial disclosure, please include your updated statement in your cover letter. Guidelines for resubmitting your figure files are available below the reviewer comments at the end of this letter.

If applicable, we recommend that you deposit your laboratory protocols in protocols.io to enhance the reproducibility of your results. Protocols.io assigns your protocol its own identifier (DOI) so that it can be cited independently in the future. For instructions see: https://journals.plos.org/plosone/s/submission-guidelines#loc-laboratory-protocols. Additionally, PLOS One offers an option for publishing peer-reviewed Lab Protocol articles, which describe protocols hosted on protocols.io. Read more information on sharing protocols at https://plos.org/protocols?utm_medium=editorial-email&utm_source=authorletters&utm_campaign=protocols.

As the corresponding author, your ORCID iD is verified in the submission system and will appear in the published article. PLOS supports the use of ORCID, and we encourage all coauthors to register for an ORCID iD and use it as well. Please encourage your coauthors to verify their ORCID iD within the submission system before final acceptance, as unverified ORCID iDs will not appear in the published article. *Only*  the individual author can complete the verification step; PLOS staff *cannot*  verify ORCID iDs on behalf of authors.

We look forward to receiving your revised manuscript.

Kind regards,

Chong Jiang

Academic Editor

PLOS One

Journal Requirements:

If the reviewer comments include a recommendation to cite specific previously published works, please review and evaluate these publications to determine whether they are relevant and should be cited. There is no requirement to cite these works unless the editor has indicated otherwise.

Please review your reference list to ensure that it is complete and correct. If you have cited papers that have been retracted, please include the rationale for doing so in the manuscript text, or remove these references and replace them with relevant current references. Any changes to the reference list should be mentioned in the rebuttal letter that accompanies your revised manuscript. If you need to cite a retracted article, indicate the article’s retracted status in the References list and also include a citation and full reference for the retraction notice.

Additional Editor Comments:

Dear Authors,

Thank you for submitting the revised version of your manuscript, “Predicting lymph node metastasis in non-small cell lung cancer combining clinical and PET/CT features with machine learning: an interpretable SHAP-based study.”

The manuscript has been substantially improved. The previous major methodological concerns have been satisfactorily addressed, particularly the information-leakage issue, test-set independence, pathological reference standard, model-selection procedure, statistical comparisons, and moderation of the clinical claims.

The revised leakage-controlled workflow and the more cautious interpretation of the moderate test-set performance considerably strengthen the manuscript. The remaining issues are limited and can be addressed in a focused minor revision.

Please provide a detailed point-by-point response to all reviewers.

Sincerely,

Chong Jiang

Academic Editor

PLOS ONE

## Reviewer 1

I appreciate the authors’ substantial revision. My main concerns in the previous round were related to information leakage, model selection using the test set, unclear pathological reference standards, and inconsistent statistical results. The authors have now rebuilt the analysis using a leakage-controlled workflow and have substantially moderated the clinical claims. I believe that the major methodological concerns have been adequately addressed. I have only a few remaining comments.

1. The final model contains only four binary predictors: central tumor location, elevated CEA, elevated SCC antigen, and solid nodule status. None of the quantitative PET parameters, including SUVmax, SUVmean, MTV, or TLG, remained in the final model. The title and Discussion should therefore avoid giving the impression that PET metabolic information provides independent predictive value. Please clarify that the final imaging contribution is mainly based on morphological features obtained from the PET/CT examination.

2. Since the final model contains only four binary variables, I suggest adding a conventional multivariable logistic regression model using exactly the same predictors as a simple reference model. Comparing its AUC, calibration, and Brier score with XGBoost would help determine whether the additional machine-learning complexity provides meaningful benefit.

3. The calibration analysis could be reported more clearly. In addition to the Brier score and calibration plot, please consider providing the calibration intercept and slope. The high NPV should also be interpreted in the context of the relatively low LNM prevalence and should not be taken as evidence that the model can safely exclude nodal metastasis in clinically node-negative patients.

4. Please continue to distinguish an internal held-out test cohort from true external validation. The current results are encouraging but remain single-center. In addition, the wide confidence interval for solid nodule status suggests some instability of this predictor, and this should be acknowledged when discussing SHAP importance and clinical interpretation.

5. The Discussion would benefit from a more up-to-date comparison with recent multicenter imaging studies of lymph-node metastasis. I suggest that the authors consider recent CT studies such as DOI: 10.1016/j.acra.2025.06.001 and DOI: 10.21147/j.issn.1000-9604.2025.01.02. Related methodological work may also be useful, particularly DOI: 10.1186/s12916-026-04865-1 and DOI: 10.1007/s00259-025-07090-9.

Overall, the revised manuscript is considerably stronger than the original submission. The authors have satisfactorily addressed my major methodological concerns, and I would support publication after these minor revisions.

---

## Reviewer 2

The authors have carefully revised the manuscript and reanalyzed the data using a leakage-free workflow. The main methodological concerns raised in the previous review have been adequately addressed, and the revised manuscript now presents the findings in a more balanced and cautious manner.

I have only a few minor comments.

1. The response states that there were no missing values and that neither imputation nor complete-case exclusion was required. Please add this information clearly to the Methods section.

2. The response also explains that CEA and SCC were recorded as normal or elevated according to the institutional laboratory reference ranges, and that the original continuous values were not available. This should also be stated in the manuscript.

3. In the Discussion, please make it clear that the added value of this model over conventional clinical and PET/CT nodal staging cannot be assessed from the present dataset, since standardized clinical N category and node-level PET/CT assessment were not available.

[Note: HTML markup is below. Please do not edit.]

Reviewers' comments:

Reviewer's Responses to Questions

**Comments to the Author**

Reviewer #1: (No Response)

Reviewer #2: All comments have been addressed

**********

2. Is the manuscript technically sound, and do the data support the conclusions?

Reviewer #1: Yes

Reviewer #2: Yes

**********

3. Has the statistical analysis been performed appropriately and rigorously?

Reviewer #1: Yes

Reviewer #2: Yes

**********

4. Have the authors made all data underlying the findings in their manuscript fully available?

The PLOS Data policy

Reviewer #1: Yes

Reviewer #2: Yes

**********

5. Is the manuscript presented in an intelligible fashion and written in standard English?

Reviewer #1: Yes

Reviewer #2: Yes

**********

Reviewer #1: I appreciate the authors’ substantial revision. My main concerns in the previous round were related to information leakage, model selection using the test set, unclear pathological reference standards, and inconsistent statistical results. The authors have now rebuilt the analysis using a leakage-controlled workflow and have substantially moderated the clinical claims. I believe that the major methodological concerns have been adequately addressed. I have only a few remaining comments.

1. The final model contains only four binary predictors: central tumor location, elevated CEA, elevated SCC antigen, and solid nodule status. None of the quantitative PET parameters, including SUVmax, SUVmean, MTV, or TLG, remained in the final model. The title and Discussion should therefore avoid giving the impression that PET metabolic information provides independent predictive value. Please clarify that the final imaging contribution is mainly based on morphological features obtained from the PET/CT examination.

2. Since the final model contains only four binary variables, I suggest adding a conventional multivariable logistic regression model using exactly the same predictors as a simple reference model. Comparing its AUC, calibration, and Brier score with XGBoost would help determine whether the additional machine-learning complexity provides meaningful benefit.

3. The calibration analysis could be reported more clearly. In addition to the Brier score and calibration plot, please consider providing the calibration intercept and slope. The high NPV should also be interpreted in the context of the relatively low LNM prevalence and should not be taken as evidence that the model can safely exclude nodal metastasis in clinically node-negative patients.

4. Please continue to distinguish an internal held-out test cohort from true external validation. The current results are encouraging but remain single-center. In addition, the wide confidence interval for solid nodule status suggests some instability of this predictor, and this should be acknowledged when discussing SHAP importance and clinical interpretation.

5. The Discussion would benefit from a more up-to-date comparison with recent multicenter imaging studies of lymph-node metastasis. I suggest that the authors consider recent CT studies such as DOI: 10.1016/j.acra.2025.06.001 and DOI: 10.21147/j.issn.1000-9604.2025.01.02. Related methodological work may also be useful, particularly DOI: 10.1186/s12916-026-04865-1 and DOI: 10.1007/s00259-025-07090-9.

Overall, the revised manuscript is considerably stronger than the original submission. The authors have satisfactorily addressed my major methodological concerns, and I would support publication after these minor revisions.

Reviewer #2: The authors have carefully revised the manuscript and reanalyzed the data using a leakage-free workflow. The main methodological concerns raised in the previous review have been adequately addressed, and the revised manuscript now presents the findings in a more balanced and cautious manner.

I have only a few minor comments.

The response states that there were no missing values and that neither imputation nor complete-case exclusion was required. Please add this information clearly to the Methods section.

The response also explains that CEA and SCC were recorded as normal or elevated according to the institutional laboratory reference ranges, and that the original continuous values were not available. This should also be stated in the manuscript.

In the Discussion, please make it clear that the added value of this model over conventional clinical and PET/CT nodal staging cannot be assessed from the present dataset, since standardized clinical N category and node-level PET/CT assessment were not available.

**********

what does this mean?). If published, this will include your full peer review and any attached files.

If you choose “no”, your identity will remain anonymous but your review may still be made public.

**Do you want your identity to be public for this peer review?**  For information about this choice, including consent withdrawal, please see our Privacy Policy

Reviewer #1: No

Reviewer #2: No

**********

[NOTE: If reviewer comments were submitted as an attachment file, they will be attached to this email and accessible via the submission site. Please log into your account, locate the manuscript record, and check for the action link "View Attachments". If this link does not appear, there are no attachment files.]

To ensure your figures meet our technical requirements, please review our figure guidelines: https://journals.plos.org/plosone/s/figures

You may also use PLOS’s free figure tool, NAAS, to help you prepare publication quality figures: https://journals.plos.org/plosone/s/figures#loc-tools-for-figure-preparation.

NAAS will assess whether your figures meet our technical requirements by comparing each figure against our figure specifications.

# Author comment: Author response to Decision Letter 2

**DOI:** 10.1371/journal.pone.0361043.r004

15 Sep 2026

Response to Reviewers

Manuscript ID: PONE-D-26-39894R1

Title: Predicting lymph node metastasis in non-small cell lung cancer using clinical and PET/CT-derived morphological features: an interpretable machine-learning study

Dear Academic Editor and Reviewers,

We thank you for the careful evaluation of our revised manuscript and for the focused suggestions for minor revision. We have addressed every comment. The principal changes include clarification that the final imaging predictors are morphological rather than quantitative PET metabolic parameters; addition of a conventional multivariable logistic regression reference model using the same four predictors; reporting of calibration intercepts and slopes; more cautious interpretation of the negative predictive value; clearer description of the internal held-out test cohort and single-center limitations; and inclusion of recent relevant studies. We also added the requested information on missing data and the coding of CEA and SCC antigen. All changes are shown in red bold text in the marked manuscript.

Response to Reviewer 1

Comment 1

The final model contains only four binary predictors: central tumor location, elevated CEA, elevated SCC antigen, and solid nodule status. None of the quantitative PET parameters, including SUVmax, SUVmean, MTV, or TLG, remained in the final model. The title and Discussion should therefore avoid giving the impression that PET metabolic information provides independent predictive value. Please clarify that the final imaging contribution is mainly based on morphological features obtained from the PET/CT examination.

Response: We agree. We revised the title and clarified in the Abstract, Results, Discussion, and Conclusion that SUVmax, SUVmean, MTV, and TLG were evaluated but were not retained after multivariable adjustment. The final imaging contribution therefore comes primarily from CT-based morphological characteristics obtained during the PET/CT examination, especially tumor location and solid nodule status, rather than from independent quantitative PET metabolic information.

Changes in the manuscript: Title; Abstract; Results, Model Performance; Discussion, first paragraph; and Conclusion. The revised title now refers to ‘clinical and PET/CT-derived morphological features.’

Comment 2

Since the final model contains only four binary variables, I suggest adding a conventional multivariable logistic regression model using exactly the same predictors as a simple reference model. Comparing its AUC, calibration, and Brier score with XGBoost would help determine whether the additional machine-learning complexity provides meaningful benefit.

Response: We performed the requested reference analysis using an unpenalized multivariable logistic regression model containing exactly the same four fixed predictors, without further feature selection or oversampling. Training performance was estimated by repeated stratified five-fold cross-validation with three repeats. The final logistic model was then fitted to the complete original training cohort and applied without modification to the internal held-out test cohort. Logistic regression achieved a test AUC of 0.733 (95% CI, 0.612–0.841), compared with 0.747 (95% CI, 0.631–0.846) for XGBoost. The paired DeLong comparison was not significant (P=0.301). The test Brier score was 0.152 for logistic regression and 0.198 for XGBoost. These findings provide no clear evidence that the additional complexity of XGBoost improved performance in this dataset.

Changes in the manuscript: Methods, Model Development and Statistical Analysis; Results, Model Performance; Table 3; Discussion, first paragraph; Conclusion; and S1 Fig.

Comment 3

The calibration analysis could be reported more clearly. In addition to the Brier score and calibration plot, please consider providing the calibration intercept and slope. The high NPV should also be interpreted in the context of the relatively low LNM prevalence and should not be taken as evidence that the model can safely exclude nodal metastasis in clinically node-negative patients.

Response: We added calibration intercepts and slopes with bootstrap 95% confidence intervals. In the internal held-out test cohort, the XGBoost calibration intercept was −1.140 (95% CI, −1.946 to −0.628) and the slope was 1.031 (95% CI, 0.568–1.930). For logistic regression, the intercept was 0.207 (95% CI, −0.649 to 1.278) and the slope was 1.095 (95% CI, 0.612–2.109). We also added S1 Fig to compare calibration graphically. In the Discussion, we now state that the XGBoost NPV of 0.905 is influenced by the test-cohort LNM prevalence of 23.4% and must not be interpreted as evidence that the model can safely exclude nodal metastasis in clinically node-negative patients.

Changes in the manuscript: Methods, Model Development and Statistical Analysis; Results, Model Performance; Discussion, first paragraph; and S1 Fig.

Comment 4

Please continue to distinguish an internal held-out test cohort from true external validation. The current results are encouraging but remain single-center. In addition, the wide confidence interval for solid nodule status suggests some instability of this predictor, and this should be acknowledged when discussing SHAP importance and clinical interpretation.

Response: We revised the manuscript to use the term ‘internal held-out test cohort’ consistently and explicitly state that the study was retrospective and single-center and that no external validation was performed. We also added a caution that the wide confidence interval for solid nodule status (OR, 9.26; 95% CI, 1.11–77.10) indicates uncertainty in the stability of this predictor. Its leading SHAP ranking is therefore interpreted as a contribution within the fitted model, not as definitive evidence of a stable or causal clinical effect.

Changes in the manuscript: Abstract; Methods, Data Processing and Model Development; Results, Model Performance and Interpreting the Model; Discussion, limitations; and Conclusion.

Comment 5

The Discussion would benefit from a more up-to-date comparison with recent multicenter imaging studies of lymph-node metastasis. I suggest that the authors consider recent CT studies such as DOI: 10.1016/j.acra.2025.06.001 and DOI: 10.21147/j.issn.1000-9604.2025.01.02. Related methodological work may also be useful, particularly DOI: 10.1186/s12916-026-04865-1 and DOI: 10.1007/s00259-025-07090-9.

Response: We reviewed all four suggested articles and found them relevant. The two recent multicenter CT studies are now discussed as direct examples of imaging-based LNM prediction with external validation. The two multimodal PET/CT studies in other malignancies are cited as related methodological work on explainable imaging-clinical integration. We have avoided direct numerical comparison because the populations, outcomes, and feature sets differ from those in our study.

Changes in the manuscript: Discussion, second paragraph; new References 18–20.

Response to Reviewer 2

Comment 1

The response states that there were no missing values and that neither imputation nor complete-case exclusion was required. Please add this information clearly to the Methods section.

Response: We added this information explicitly. No values were missing for the variables included in the analyses; therefore, neither data imputation nor complete-case exclusion was required.

Changes in the manuscript: Methods, Data Processing.

Comment 2

The response also explains that CEA and SCC were recorded as normal or elevated according to the institutional laboratory reference ranges, and that the original continuous values were not available. This should also be stated in the manuscript.

Response: We added this information to the Methods. CEA and SCC antigen were recorded as normal or elevated according to the institutional laboratory reference ranges applicable at the time of measurement, and the original continuous concentrations were not available for this analysis.

Changes in the manuscript: Methods, Patients and variable collection.

Comment 3

In the Discussion, please make it clear that the added value of this model over conventional clinical and PET/CT nodal staging cannot be assessed from the present dataset, since standardized clinical N category and node-level PET/CT assessment were not available.

Response: We agree and have added this limitation explicitly. Because standardized clinical N category and node-level PET/CT assessments were unavailable, the incremental value of the model beyond conventional clinical and PET/CT nodal staging could not be evaluated. The manuscript also avoids claiming that the model can replace conventional staging or safely exclude metastasis in clinically node-negative patients.

Changes in the manuscript: Discussion, limitations; Conclusion.

We again thank the Academic Editor and Reviewers for their constructive comments, which helped us improve the clarity and balance of the manuscript.

Sincerely,

The Authors

Attachment Submitted filename: Response_to_Reviewers.docx

# Peer review: Decision Letter 2

**DOI:** 10.1371/journal.pone.0361043.r005

**Authors:** Carmelo Caldarella

6 Oct 2026

Predicting lymph node metastasis in non-small cell lung cancer using clinical and PET/CT-derived morphological features: an interpretable machine-learning study

PONE-D-26-39894R2

Dear Dr. Xu,

We’re pleased to inform you that your manuscript has been judged scientifically suitable for publication and will be formally accepted for publication once it meets all outstanding technical requirements.

Within one week, you’ll receive an e-mail detailing the required amendments. When these have been addressed, you’ll receive a formal acceptance letter and your manuscript will be scheduled for publication.

An invoice will be generated when your article is formally accepted. Please note, if your institution has a publishing partnership with PLOS and your article meets the relevant criteria, all or part of your publication costs will be covered. Please make sure your user information is up-to-date by logging into Editorial Manager at Editorial Manager® and clicking the ‘Update My Information' link at the top of the page. For questions related to billing, please contact billing support.

If your institution or institutions have a press office, please notify them about your upcoming paper to help maximize its impact. If they’ll be preparing press materials, please inform our press team as soon as possible -- no later than 48 hours after receiving the formal acceptance. Your manuscript will remain under strict press embargo until 2 pm Eastern Time on the date of publication. For more information, please contact onepress@plos.org.

Kind regards,

Carmelo Caldarella, Ph.D., M.D.

Academic Editor

PLOS One

Additional Editor Comments (optional):

Reviewers' comments:

Reviewer's Responses to Questions

**Comments to the Author**

Reviewer #1: All comments have been addressed

Reviewer #2: All comments have been addressed

**********

2. Is the manuscript technically sound, and do the data support the conclusions?

Reviewer #1: Yes

Reviewer #2: Yes

**********

3. Has the statistical analysis been performed appropriately and rigorously?

Reviewer #1: Yes

Reviewer #2: Yes

**********

4. Have the authors made all data underlying the findings in their manuscript fully available?

The PLOS Data policy

Reviewer #1: Yes

Reviewer #2: Yes

**********

5. Is the manuscript presented in an intelligible fashion and written in standard English?

Reviewer #1: Yes

Reviewer #2: Yes

**********

Reviewer #1: Thank you for the careful revision. The authors have adequately addressed my previous comments and have substantially improved the clarity and methodological transparency of the manuscript. In particular, the additional comparison with conventional logistic regression, the expanded calibration analysis, and the more cautious interpretation of the model’s clinical utility have strengthened the manuscript. The limitations of the single-center internal validation are also now appropriately acknowledged. I have no further substantive concerns and consider the manuscript suitable for publication.

Reviewer #2: The authors have adequately addressed the comments raised in the previous round of review. In particular, the manuscript now clearly describes the handling of missing data, the coding of CEA and SCC antigen, and the limitation regarding the lack of standardized clinical N staging and node-level PET/CT assessment.

The revisions have improved the clarity and balance of the manuscript, and I have no further comments.

I believe the manuscript is suitable for publication in its current form.

**********

what does this mean?). If published, this will include your full peer review and any attached files.

If you choose “no”, your identity will remain anonymous but your review may still be made public.

**Do you want your identity to be public for this peer review?**  For information about this choice, including consent withdrawal, please see our Privacy Policy

Reviewer #1: No

Reviewer #2: No

**********

# Editor report: Acceptance letter

**DOI:** 10.1371/journal.pone.0361043.r006

**Authors:** Carmelo Caldarella

PONE-D-26-39894R2

PLOS One

Dear Dr. Xu,

I'm pleased to inform you that your manuscript has been deemed suitable for publication in PLOS One. Congratulations! Your manuscript is now being handed over to our production team.

At this stage, our production department will prepare your paper for publication. This includes ensuring the following:

* All references, tables, and figures are properly cited

* All relevant supporting information is included in the manuscript submission,

* There are no issues that prevent the paper from being properly typeset

You will receive further instructions from the production team, including instructions on how to review your proof when it is ready. Please keep in mind that we are working through a large volume of accepted articles, so please give us a few days to review your paper and let you know the next and final steps.

Lastly, if your institution or institutions have a press office, please let them know about your upcoming paper now to help maximize its impact. If they'll be preparing press materials, please inform our press team within the next 48 hours. Your manuscript will remain under strict press embargo until 2 pm Eastern Time on the date of publication. For more information, please contact onepress@plos.org.

You will receive an invoice from PLOS for your publication fee after your manuscript has reached the completed accept phase. If you receive an email requesting payment before acceptance or for any other service, this may be a phishing scheme. Learn how to identify phishing emails and protect your accounts at https://explore.plos.org/phishing.

If we can help with anything else, please email us at customercare@plos.org.

Thank you for submitting your work to PLOS One and supporting open access.

Kind regards,

PLOS One Editorial Office Staff

on behalf of

Dr. Carmelo Caldarella

Academic Editor

PLOS One
